# Chemical synthesis, characterisation and in vitro and in vivo metabolism of the synthetic opioid MT-45 and its newly identified fluorinated analogue 2F-MT-45 with metabolite confirmation in urine samples from known drug users

**DOI:** 10.1007/s11419-018-0413-1

**Published:** 2018-04-05

**Authors:** Craig McKenzie, Oliver B. Sutcliffe, Kevin D. Read, Paul Scullion, Ola Epemolu, Daniel Fletcher, Anders Helander, Olof Beck, Alexia Rylski, Lysbeth H. Antonides, Jennifer Riley, Shannah A. Smith, Niamh Nic Daeid

**Affiliations:** 1 Forensic Drug Research Centre, Centre for Anatomy and Human Identification, School of Science and Engineering, University of Dundee, Dundee, UK; 2 Division of Chemistry and Environmental Science, School of Science and the Environment, Manchester Metropolitan University, Manchester, UK; 3 Drug Discovery Unit, School of Life Sciences, University of Dundee, Dundee, UK; 4 Department of Laboratory Medicine, Karolinska Institutet and Karolinska University Laboratory, Stockholm, Sweden; 5 Leverhulme Research Centre for Forensic Science, School of Science and Engineering, University of Dundee, Dundee, UK

**Keywords:** Novel psychoactive substances, MT-45, 2F-MT-45, Metabolite identification, Synthetic opioids, Clinical and forensic toxicology

## Abstract

**Purpose:**

The detection of a novel psychoactive substance, 2F-MT-45, a fluorinated analogue of the synthetic opioid MT-45, was reported in a single seized tablet. MT-45, 2F-, 3F- and 4F-MT-45 were synthesised and reference analytical data were reported. The in vitro and in vivo metabolisms of MT-45 and 2F-MT-45 were investigated.

**Method:**

The reference standards and seized sample were characterised using nuclear magnetic resonance spectroscopy, ultra-performance liquid chromatography–quadrupole time of flight mass spectrometry, gas chromatography–mass spectrometry, attenuated total reflectance-Fourier transform infrared spectroscopy and Raman spectroscopy. Presumptive tests were performed and physicochemical properties of the compounds determined. Metabolite identification studies using human liver microsomes, human hepatocytes, mouse hepatocytes and in vivo testing using mice were performed and identified MT-45 metabolites were confirmed in authentic human urine samples.

**Results:**

Metabolic pathways identified for MT-45 and 2F-MT-45 were *N*-dealkylation, hydroxylation and subsequent glucuronidation. The major MT-45 metabolites identified in human in vitro studies and in authenticated human urine were phase I metabolites and should be incorporated as analytical targets to existing toxicological screening methods. Phase II glucuronidated metabolites were present in much lower proportions.

**Conclusions:**

2F-MT-45 has been detected in a seized tablet for the first time. The metabolite identification data provide useful urinary metabolite targets for forensic and clinical testing for MT-45 and allows screening of urine for 2F-MT-45 and its major metabolites to determine its prevalence in case work.

**Electronic supplementary material:**

The online version of this article (10.1007/s11419-018-0413-1) contains supplementary material, which is available to authorized users.

## Introduction

**Fig. 1:**
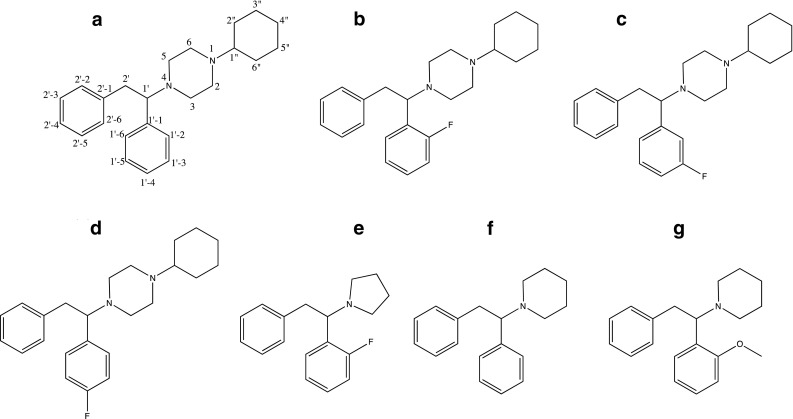
Chemical structures of **a** MT-45, including the structural notation used for nuclear magnetic resonance (NMR) spectroscopy data using the system reported by [16], **b** 2F-MT-45, **c** 3F-MT-45, **d** 4F-MT-45, **e** 2FPPP (fluorolintane), **f** diphenidine and **g** 2-methoxphenidine (2-MXP)

MT-45 [1-cyclohexyl-4-(1,2-diphenylethyl)piperazine], also known as IC-6, NSC 299236 and CDEP, is a synthetic opioid developed as a therapeutic drug candidate in the 1970s by the Dainippon Pharmaceutical Co. in Japan. MT-45 has never been commercially available as a therapeutic agent; however, it has appeared on the illicit recreational drug market in recent years, but not extensively [1–6]. MT-45 is structurally distinct from other therapeutic opioids (Fig. 1a) and has structural similarities to the dissociative drug diphenidine (Fig. 1f) which is an *N*-methyl-d-aspartic acid (NMDA) receptor agonist. MT-45 is a selective μ-opioid receptor (MOR-1) agonist showing nM binding affinities to MOR-1, but is less potent than morphine, with considerably lower *δ*- and *κ*-opioid receptor (DOR-1 and KOR-1) affinities [1]. The racemic mixture has opioid-like properties in animal models with a potency similar to morphine, with the (*S*)-enantiomer being considerably more potent than the (*R*)-isomer [4, 7–11]. Antinociceptive (reduced sensitivity to pain) effects were studied in vivo using a radiant heat tail flick assay in mice, showing that MT-45 had analgesic properties similar to morphine [1], which were further confirmed by Montesano et al. [12]. Users reporting their subjective experiences with the drug on online fora have often reported dissociative effects after consuming MT-45 [13–15], and such effects may be mediated via the NMDA receptor; however, no formal studies on the NMDA receptor affinity of MT-45 or its metabolites have been reported.

MT-45 was first detected on the illicit market in seized samples in Japan [16] and was first reported in Europe by the STRIDA project in Sweden in nine nonfatal intoxication cases [17]. Detection of MT-45 in biological fluids was reported to the European Monitoring Centre for Drugs and Drug Addiction (EMCDDA) in relation to 28 fatal and 12 nonfatal intoxications in Sweden, the substance being implicated in 19 of the fatalities either as the cause of death or as a contributing factor and was commonly detected with other psychoactive substances [18]. Unusual side effects, including hearing impairment and loss, severe bilateral cataracts, folliculitis and dermatitis were reported in individuals following nonfatal intoxications with MT-45 [17, 19, 20]. In the USA, MT-45 and the benzodiazepine etizolam were detected and quantified in whole femoral blood relating to a drug-related fatality (520 and 35 ng/mL, respectively), and MT-45 was also detected in urine, vitreous humor and bile [21]. Two further MT-45-related fatalities were reported in the USA in 2013 [22]. MT-45 was detected (2900 ng/mL) in postmortem femoral blood in a German fatality, where the user appeared to die shortly after taking the drug [23]. Both Papsun et al. [21] and Fels et al. [23] noted that MT-45 in femoral blood appeared unstable (and of unknown stability in other matrices) with concentrations decreasing > 50% from original concentrations over approximately 1 year, despite samples being stored at −20 °C until analysis; however, the breakdown products of MT-45 have not been identified. Reviews of the scientific literature related to MT-45 [2, 24–26] highlight a lack of availability of metabolic studies, although one such study has recently been published providing in vitro data using rat hepatocytes and in vivo data in mice [12].

A review of the prevalence of MT-45, its mode of use and user experiences taken from online fora is available, together with a report on MT-45 anecdotally being seen as a relatively undesirable recreational drug [27, 28]. In the UK, MT-45 was designated a Class A substance under the Misuse of Drugs Act 1971 (as amended) in February 2015 and is a Schedule 1 substance in the Misuse of Drugs Regulations 2001 (as amended) having no legitimate medical application [24]. In May 2016, MT-45 was brought under international control by the United Nations Office for Drugs and Crime (UNODC) as a Schedule I substance under the Single Convention on Narcotic Drugs of 1961, as amended by the 1972 Protocol [25]. In July 2017, it was listed as a controlled substance by China, prohibiting its sale and export, one of 138 substances controlled in this way since 2015 [29]. One common response of novel psychoactive substance (NPS) drug synthesis laboratories supplying the illicit drug market, most commonly, but not exclusively, located in China, following a legislative ban, is to manipulate the structure of the banned substance to a small degree, evading the ban but retaining similar effects and potency. One such manipulation is the substitution of a hydrogen atom on the molecule with a fluorine atom. It might be expected therefore that, following the ban, fluorinated analogues of MT-45 may appear on the illicit drug market.

This study reports the first known identification of a fluorinated MT-45 analogue, detected in a tablet recovered in Manchester, UK, in October 2016, and describes the substance’s analytical characterization and the synthesis of three fluorinated MT-45 analogue reference standards (2F-, 3F- and 4F-MT-45) to unequivocally confirm identity. This study also reports the elucidation and identification of phase I and II metabolites of MT-45 and, for the first time, 2F-MT-45. The in vitro and in vivo metabolisms of MT-45 and 2F-MT-45 have been studied using human liver microsomes, human hepatocytes, mouse hepatocytes and mouse in vivo testing, and the data from the different techniques are compared as well to previously published MT-45 metabolite identification data [12]. MT-45 metabolites identified in vitro and mouse in vivo studies have, for the first time, been analytically confirmed in reanalysed human urine samples from individuals known to have ingested MT-45.

## Materials and methods

### Synthesis of reference standards

1-Cyclohexyl-4-(1,2-diphenylethyl)piperazine dihydrochloride (MT-45, Fig. 1a) and its fluorinated derivatives (2F-MT-45, Fig. 1b; 3F-MT-45, Fig. 1c; 4F-MT-45, Fig. 1d) were prepared using an adaptation of the method reported by Geyer et al. [30]. The products were structurally characterized by ^1^H nuclear magnetic resonance (NMR), ^13^C NMR, ^19^F NMR, gas chromatography–mass spectrometry (GC–MS), attenuated total reflectance-Fourier transform infrared (ATR-FTIR) spectroscopy and Raman spectroscopy (see supplementary material). Yields of products (after recrystallization from diethyl ether): 1-cyclohexyl-4-(1,2-diphenylethyl)piperazine dihydrochloride (MT-45, 20%); 1-cyclohexyl-4-(1-(2-fluorophenyl)-2-phenylethyl)piperazine dihydrochloride (2F-MT-45, 18%); 1-cyclohexyl-4-(1-(3-fluorophenyl)-2-phenylethyl)piperazine dihydrochloride (3F-MT-45, 19%); 1-cyclohexyl-4-(1-(4-fluorophenyl)-2-phenylethyl)piperazine dihydrochloride (4F-MT-45, 18%).

### Seized illicit tablet

**Fig. 2:**
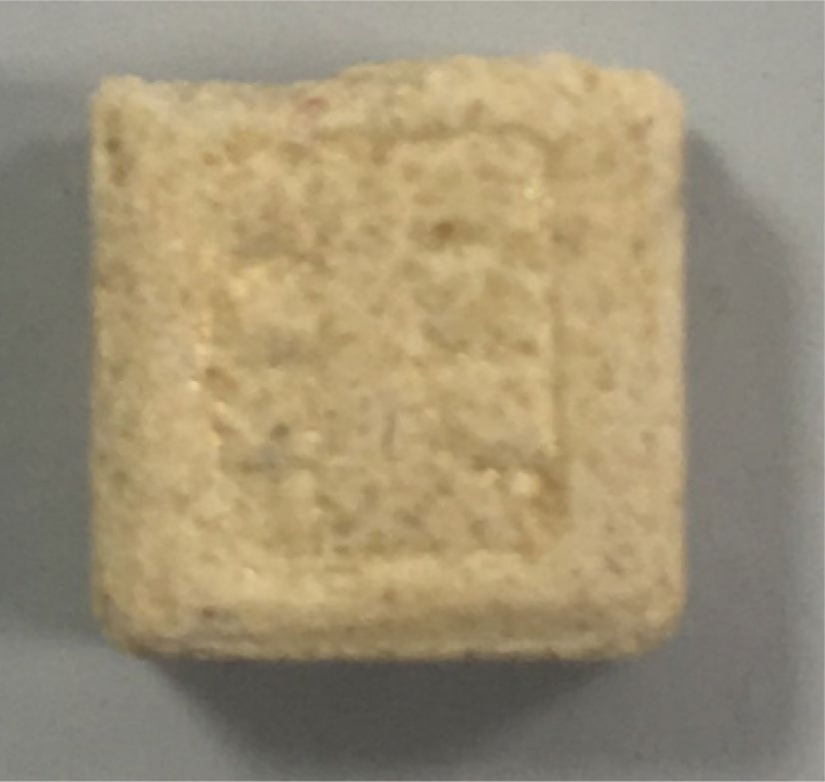
Photograph of off-white/cream square tablet (in a clear snap-bag labelled with “2FPPP”), with 6-mm length, 6-mm width and 4-mm height/depth

An off-white/cream square tablet in a clear snap-bag labelled with “2FPPP”, an alternative name for the dissociative drug fluorolintane, was submitted to Manchester Metropolitan University via Greater Manchester Police on the 15th October 2016 following the Mantra Festival, Greater Manchester, UK. The tablet was 6 mm in length × 6 mm width × 4 mm in height/depth. The molecular structure of 2FPPP is shown in Fig. 1e and the seized tablet is shown in Fig. 2.

### Chemical characterisation

GC–MS analysis was carried out on 1-mg/mL sample analyte solutions in high-performance liquid chromatography (HPLC) grade methanol using a 7820A gas chromatograph coupled to a 5977E mass spectrometer (Agilent Technologies, Santa Clara, CA, USA). Injection mode: 1-μL sample injection and 25:1 split, injection port temperature: 250 ^°^C, carrier gas: He, flow: 1 mL/min. Column: DB-1MS, 25 m × 0.2 mm i.d.,  film thickness 0.33 μm (Agilent Technologies). GC oven: 40 ^°^C held for 1 min; 40 ^°^C/min to 300 ^°^C held for 4 min. Transfer line: 280 ^°^C. The mass spectrometer was operated in electron ionization (EI) mode. Ionization conditions: 70 eV in full scan mode (50–550 amu), ion source: 230 ^°^C, quadrupole: 150 ^°^C.

ATR-FTIR spectroscopy was carried out using a CARY 630 system (Agilent Technologies).

NMR spectroscopy analyses were performed using a Bruker AVANCE III HD 500-MHz spectrometer (Bruker, Billerica, MA, USA) running under TopSpin v.3.2.5 equipped with a QCI-F cryo-probe at a sample compartment temperature of 25 °C. Samples were prepared in CDCl_3_ (~ 10 mg in 1 mL). Two drops of saturated sodium bicarbonate in D_2_O was added and the samples shaken thoroughly to extract the salt. Once the layers of CDCl_3_ and D_2_O had fully separated, a 0.7-mL aliquot of the CDCl_3_ layer was recovered and transferred to NMR tubes for analysis. The data was analysed using TopSpin v.3.2.3. The residual solvent signals at *δ* = 7.26 ppm for ^1^H and *δ* = 77.16 ppm for ^13^C were used as internal references. Hexafluorobenzene was used to reference the ^19^F spectra (*δ* = −164.9 ppm). Characterisation of the compound was performed using ^1^H NMR, ^13^C NMR (^1^H decoupled), ^19^F NMR (^1^H decoupled), double-quantum filtered correlated spectroscopy (COSY-DQF), 1-bond ^1^H-^13^C distortionless enhancement by polarisation transfer (DEPT)-135 edited heteronuclear single quantum correlation (HSQC) spectroscopy, ^1^H-^13^C heteronuclear multiple bond coherence (HMBC) spectroscopy, nuclear Overhauser enhancement spectroscopy (NOESY) and ^19^F-^1^H heteronuclear Overhauser enhancement spectroscopy (HOESY; see supplementary material for all spectral characterisations). The numbering scheme for the structure is shown in Fig. 1a [16].

Ultra-performance liquid chromatography–quadrupole time-of-flight mass spectrometry (UPLC–QToF-MS) of the synthesised reference standards and metabolomics samples was performed using an Acquity UPLC instrument consisting of a binary pump, autosampler (held at 4 ^°^C), vacuum degasser and column oven (held at 40 ^°^C) coupled to a Xevo-QToF (Waters Corporation, Milford, MA, USA). Mobile phases were (A) LC–MS grade water with 0.1% formic acid and (B) acetonitrile with 0.1% formic acid. The gradient used was: 0.0–0.5 min: 2% B; 0.5–5.0 min: 2–95% B; 5.0–5.99 min: 95% B; and 6.0–7.0 min: 2% B for equilibration. Flow rate was 0.5 mL/min and 2 μL of sample was injected onto a BEH C_18_ 50 × 2.1 mm, 1.7 µm particle size column (Waters Corporation). The QToF was operated in positive ionisation mode with a source temperature at 120 ^°^C, a desolvation temperature at 500 ^°^C and a capillary voltage at 2.25 kV. QToF-MS analysis was carried out with a collision energy at 6 V and QToF MS^e^ acquisition was carried out using collision energy ranging from 20 to 40 V. Once QToF-MS, and MS^e^ data were processed, MS/MS data acquisition was utilised for selected parent ion accurate mass data to determine accurate product ion data.

Raman spectroscopy was performed using a proprietary analytical system developed at the University of Dundee (see supplementary material).

The p*K*a-basic and partition coefficient (log *P*) values for MT-45 and its fluorinated analogues were experimentally derived using a Sirius T3 system (Sirius Analytical Instruments, Sussex, UK).

A range of presumptive colour tests were carried out using 1–2 mg of the synthesised reference standards and the seized tablet, with reagents prepared and tests performed in accordance with UNODC guidelines [31].

### Metabolite identification

Full method details for the in vitro metabolite identification studies are provided in the supplementary material.

#### Pooled human liver microsome incubations

MT-45, 2F-MT-45 and positive controls were incubated in pooled human liver microsome (pHLM; Thermo Fisher Scientific, Waltham, MA, USA) incubations at 37 °C with and without uridine 5′-diphosphoglucuronic acid (UDPGA). Verapamil was used as the positive control for incubations without UDPGA (phase I metabolism) and propranolol as the positive control for incubations with UDPGA (phase II metabolism). *Procedure*: 450 µL of liver microsomes (1.11 mg/mL) and 45 µL of cofactor (nicotinamide adenine dinucleotide phosphate (NADPH) or UDPGA/NADPH) were added to wells of a 96-deep-well 2-mL plate (incubation plate) kept at 37 °C; 5 µL of 5 μM test compound in dimethyl sulphoxide (DMSO; Fisher Scientific, Loughborough, UK) was added and a 50-µL aliquot of the incubation mixture was immediately removed and added to 200 µL of acetonitrile (ACN; Fisher Scientific) to terminate the reaction, providing an initial (*t *= 0) sample. Further, 50-µL aliquots of the incubation mixtures were removed at 5, 15, 30 and 60 min. All samples were centrifuged to sediment any precipitated protein (3270 rpm for 10 min). A 150-µL volume of the supernatant was removed, diluted with 50 µL of Milli-Q water in a 96 deep-well 2-mL plate (analysis plate) and sealed prior to analysis by UPLC–QToF-MS.

#### In vitro human and mouse hepatocyte incubations

Metabolic stability studies were performed using cryopreserved hepatocytes; either mouse (Mheps: mouse CD1 Cryo Hep Female Suspension Pool 20 Donor, 4–8 million recoverable cells, MSCS20; Thermo Fisher Scientific) or human (Hheps: Human Cryo Hep Suspension Pool 50 Donor, mixed gender, HMCS50; Thermo Fisher Scientific). 7-Ethoxycoumarin and 7-hydroxycoumarin were used as positive controls. *Reaction initiation and sampling:* 200 μL of working solution containing test compound at 5 μM in DMSO was added to 200 μL of cell suspension to initiate the reaction. A 20-μL aliquot of the incubation mixture was removed immediately and added to 80 μL of acetonitrile containing an internal standard (50 ng/mL donepezil). Further 20-μL aliquots were removed to a 96 deep-well 2-mL plate (analysis plate) at the following timepoints: 3, 6, 9, 15, 30, 60, 90 and 120 min. A 100-μL volume of water/acetonitrile (80:20, v/v) was added to all samples and the analysis plate was centrifuged at 2800 rpm for 10 min at room temperature prior to injection and analysis of samples by UPLC–QToF-MS.

#### In vivo mouse metabolite identification studies

MT-45 and 2F-MT-45 were dosed orally by gavage as a solution at 10 mg free base/kg (dose volume: 10 mL/kg; dose vehicle: 1.0% carboxymethyl cellulose) to female C57BJ/6 J mice (*n *= 1/compound). Blood samples (10 µL) were taken from each mouse tail vein pre-dose and then at 0.5, 1, 2, 4, 8 and 24 h, mixed with 9 volumes of distilled water and stored frozen until analysed. The 24-h urine samples were also collected for metabolite identification studies. The blood and urine samples were subjected to UPLC–QToF-MS analysis and the metabolites were identified. All samples were processed using MetaboLynx-XS software (Waters Corporation) to identify possible metabolites from MS data. The list was reduced by a manual check of the data and an MS/MS analysis was performed on peaks accepted as genuine metabolites.

#### Human metabolite identification studies

In addition to the identification of MT-45 metabolites identified in the in vitro and mouse in vivo, the study in human urinary analysis was carried out using urine samples collected from two analytically confirmed intoxication cases, two males aged 17 and 26 years (full details are provided in [17]) enrolled in the Swedish STRIDA project on NPS [17, 19]. The 17-year-old had 102 ng/mL of MT-45 in blood and 43 ng/mmol of creatinine in urine. 11-Nor-9-carboxy-∆^9^-tetrahydocannabinol, dextromethorphan and methiopropamine were also detected in his urine. The 26-year-old had 39 ng/mL of MT-45 in blood and 200 ng/mmol of creatinine in urine and 3-methoxyphencyclidine was also detected. The urine samples had been stored at −80 °C since the time of sampling to reanalysis for this study. The analysis was done by HPLC combined with high-resolution mass spectrometry (HRMS), essentially as detailed elsewhere [32, 33], and involved a targeted search for the metabolites already identified in the human and mouse in vitro and mouse in vivo studies.

## Results

### Chemical characterisation of synthesised reference standards

UPLC–QToF-MS, GC–MS, ATR-FTIR spectroscopy, NMR (^1^H, ^13^C and ^19^F) spectroscopy and Raman spectroscopy data and experimentally derived p*K*a’s, log *P* (octanol/water) and log *D*_7.4_ (octanol/water) values for the characterisation of the synthesised reference standards were provided (see supplementary material, section C). Log *P* is the partition coefficient of the non-ionised form of the analyte, whilst log *D* is the pH-dependent coefficient of distribution for ionisable compounds such as the MT-45s at the physiological pH (pH 7.4).

The MT-45 and fluorinated analogue reference standards were tested using a range of commonly used presumptive tests (see supplementary material). MT-45 and its fluorinated analogues gave similar, positive results for Scott’s (cobalt (II) thiocyanate) test, normally used as a presumptive test for cocaine, to those previously reported for the structurally similar diphenidine and 2-, 3- and 4-methoxphenidine (MXP), most likely due to the presence of tertiary amines in all [31]. However, the MT-45s did not give a positive result in the modified Scott’s test, which allows them to be differentiated from diphenidine and the MXPs [McKenzie et al., unpublished data]. The MT-45s all gave a yellow result in the Mecke’s test as did diphenidine and 2-, 3- and 4-MXP [McKenzie et al., unpublished data]. Diphenidine and the MT-45s produced similar responses to the Marquis test (immediate yellow) but were differentiated from the MXPs which produce a transient pink or red/brown reaction [30].

### Identification of seized material

The seized tablet was analysed by GC–MS, ATR-FTIR spectroscopy and NMR spectroscopy. Presumptive testing provided the same results for the seized tablet as observed for MT-45 and its fluorinated analogue reference standards. Preliminary GC–MS analysis indicated that it contained a previously unreported fluorinated MT-45 derivative rather than 2FPPP. The mass spectrum of the seized sample revealed a molecular ion at *m*/*z* 366, and a comparison to the mass spectra of the MT-45 and 2F-MT-45 reference standards is provided in the supplementary material. MT-45 could be chromatographically separated from the fluorinated MT-45s; however, the regioisomers could not be separated by retention time or mass using GC–MS. Comparison of the seized tablet with the synthesised reference standards using ATR-FTIR spectroscopy indicated that 2F-MT-45 was present with two discriminatory areas in the fingerprint region of the spectra, 800–650 nm and 1550–1400 nm (see supplementary material).

**Fig. 3:**
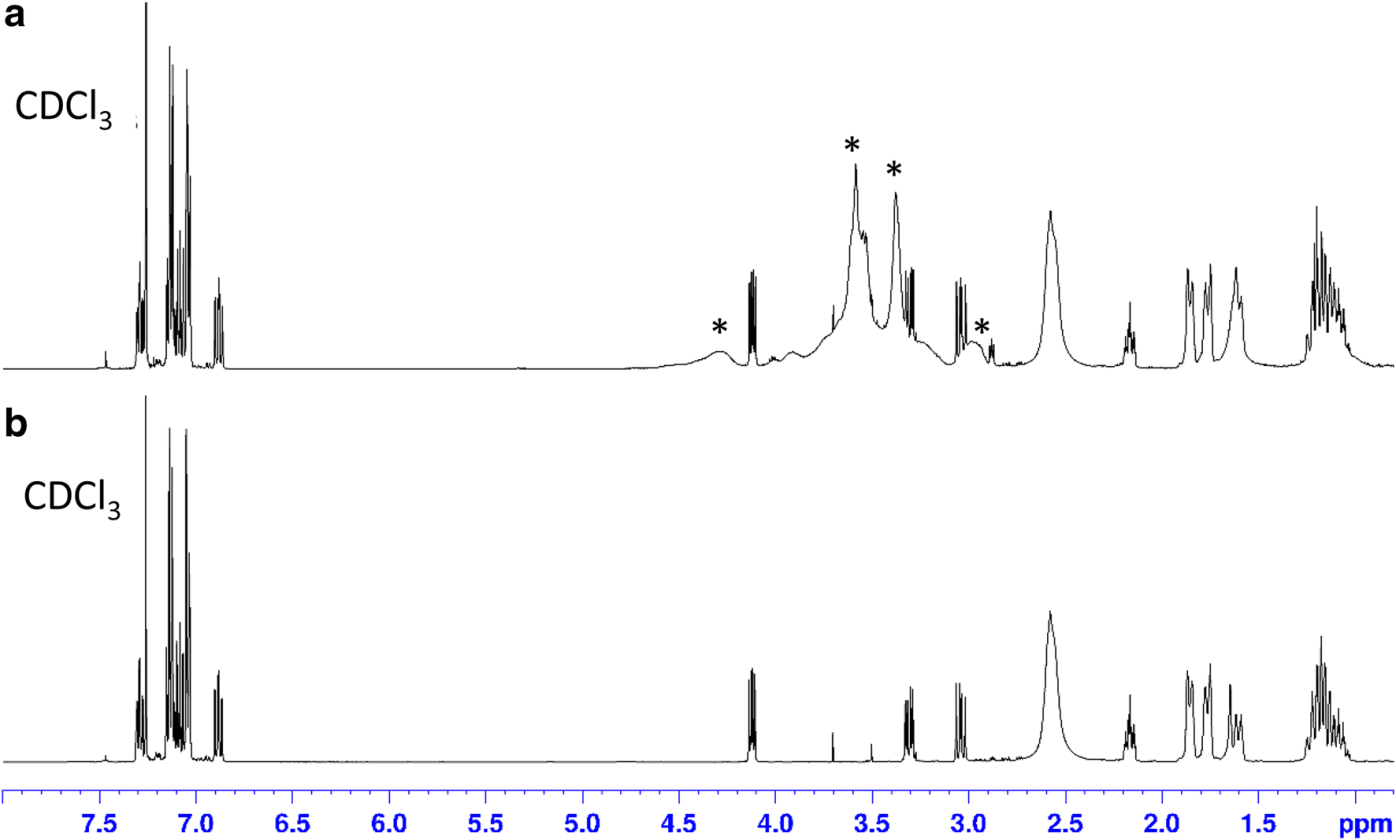
**a** ^1^H NMR data for the seized sample, and **b** ^1^H NMR data for the 2F-MT-45 reference standard. *Signals presumed to be originating from unknown excipient(s) present in the seized sample

**Fig. 4:**
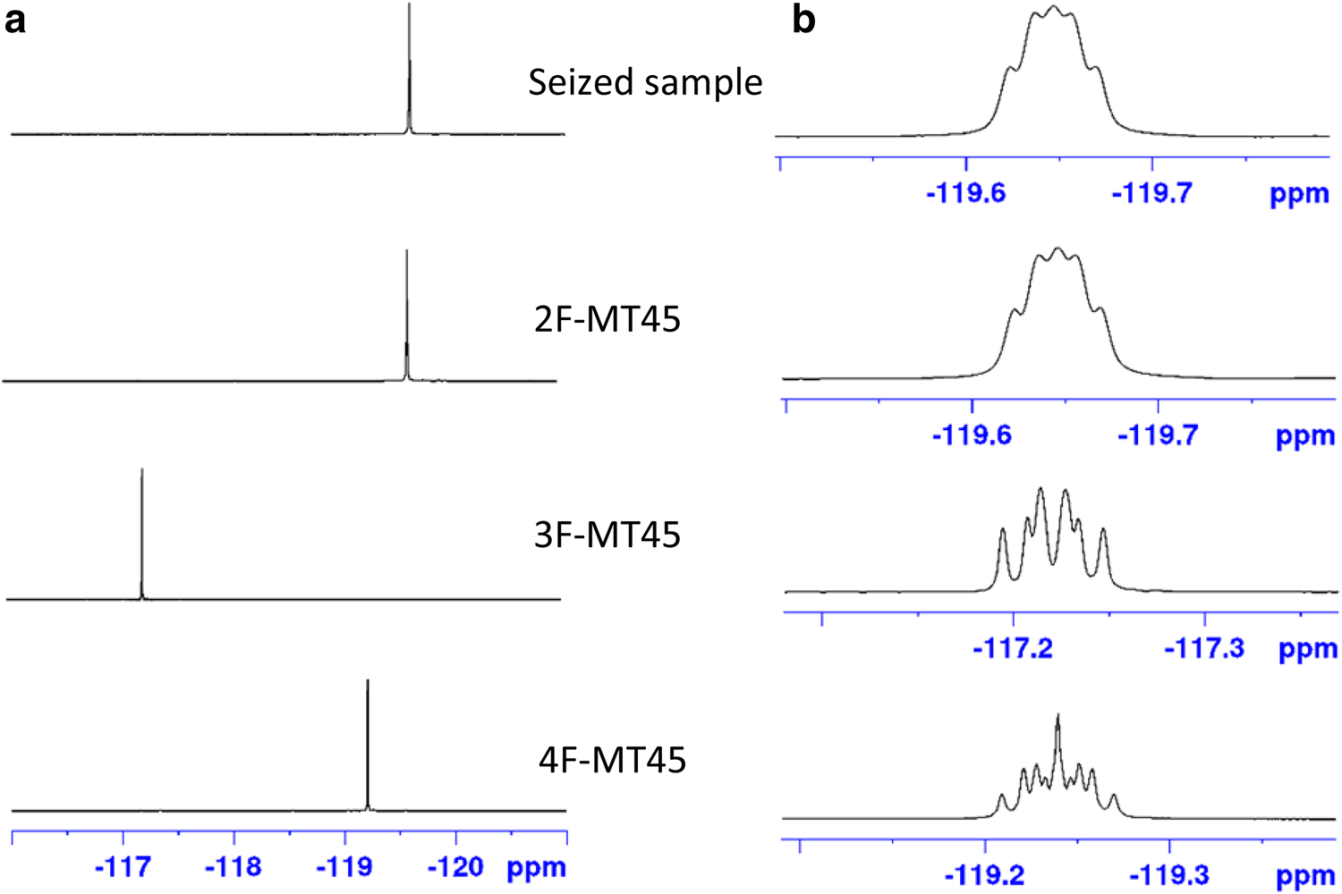
Comparison of **a** proton decoupled and **b** proton-coupled ^19^F NMR spectra for the seized tablet from bag labelled “2FPPP” and the synthesised fluorinated MT45 reference standards

NMR spectroscopy data for the seized sample unequivocally confirmed the presence of 2F-MT-45 in the seized tablet along with unidentified excipients (Table 1). The data indicated the presence of a mono-fluorinated diphenylethyl group, a piperazine group (broadened by exchange processes) and a cyclohexane group. The connections between these groups and the position of the fluorine were determined using HMBC and NOESY/HOESY data (see supplementary material). The HMBC/NOESY/HOESY correlations to/from the 1′ proton confirmed that the molecule was fluorinated at the 1′-2 position. This was further confirmed when the ^1^H NMR and ^19^F NMR data from the F-MT-45 reference standards were compared with the seized sample (Figs. 3 and 4). The position of the fluorine singlet in the decoupled spectra showed the identical signals for the 2F-MT-45 and the seized sample (both *δ:* −119.6 ppm), and the proton-coupled data provides greater localised structural information and further evidence that the seized sample contains 2F-MT-45. To the author’s knowledge, this is the first identification of a fluorinated MT-45 analogue in a seized sample.

**Table 1:** ^13^C, ^1^H and ^19^F nuclear magnetic resonance data for the seized tablet and a 2F-MT-45 reference standard (ref. std.), with a structural numbering system as used by [16] and as shown in Fig. 1a

No.	Seized sample in CDCl_3_ Chemical shifts, *δ* in ppm			2F-MT-45 ref. std. in CDCl_3_ Chemical shifts, δ in ppm		
	^1^H	^13^C	^19^F	^1^H	^13^C	^19^F
2/6	2.58, 2/6 overlaps with 3/5, total integral 8H, br	49.37, CH_2_		2.58, 2/6 overlaps with 3/5, total integral 8H, br	49.43, CH_2_	
3/5	2.54, br	50.34, br, CH_2_		2.55, br	50.42, br, CH_2_	
1′	4.12, 1H, dd, *J* = 9.6, 5.6 Hz	63.59/63.56*, CH		4.12, 1H, dd, *J* = 9.7, 5.6 Hz	63.59/63.54*, CH	
2′	3.32, 1H, dd, *J* = 13.5, 5.6 Hz	38.43, CH_2_, d, *J *= 1.2 Hz		3.31, 1H, dd, *J* = 13.5, 5.6 Hz	38.44, CH_2_, d, *J *= 1.2 Hz	
	3.04, 1H, dd, *J* = 13.5, 9.6 Hz			3.04, 1H, dd, *J* = 13.5, 9.7 Hz		
1′-1	–	125.68, C, d, *J* = 14.5 Hz		–	125.67, C, d, *J* = 14.7 Hz	
1′-2	–	161.52, C, d, *J* = 244.8 Hz	−119.6	–	161.53, C, d, *J* = 244.8 Hz	−119.6
1′-3	6.88, 1H, ddd, *J* = 10.2, 8.2, 1.2 Hz	115.42, CH, d, *J* = 23.8 Hz		6.88, 1H, ddd, *J* = 10.2, 8.2, 1.2 Hz	115.43, CH, d, *J* = 23.8 Hz	
1′-4	7.13, 1H, overlapping	128.59, CH, d, *J* = 8.5 Hz		7.13, 1H, overlapping	128.56, CH, d, *J* = 8.5 Hz	
1′-5	7.05, 1H, overlapping	123.62, CH, d, *J* = 3.4 Hz		7.05, 1H, overlapping	123.61, CH, d, *J* = 3.3 Hz	
1′-6	7.29, 1H, td, *J* = 7.3, 1.7 Hz	130.05, CH, d, *J* = 4.9 Hz		7.29, 1H, td, *J* = 7.4, 1.8 Hz	130.06, CH, d, *J* = 4.9 Hz	
2′-1	–	139.30, C		–	139.35, C	
2′-2/2′-6	7.04, 2H, overlapping	129.37, CH		7.04, 2H, overlapping	129.38, CH	
2′-3/2′-5	7.14, 2H, overlapping	128.09, CH		7.14, 2H, overlapping	128.09, CH	
2′-4	7.08, 1H	126.00, CH		7.09, 1H	126.00, CH	
1″	2.17, 1H, tt, *J *= 10.9, 3.3 Hz	63.59/63.56*, CH		2.17, 1H, tt, *J *= 10.9, 3.3 Hz	63.54/63.59*, CH	
2″/6″	1.86, 2H, brd, *J* = 12.1 Hz	29.14, 29.12, CH_2_		1.86, 2H, brd, *J* = 12.1 Hz	29.20, 29.17, CH_2_	
	1.15, 2H, m			1.15, 2H, m		
3″/5″	1.76, 2H, brd, *J* = 13.0 Hz	26.02, CH_2_		1.77, 2H, brd, *J* = 12.8 Hz	26.03, CH_2_	
	1.19, 2H, m			1.21, 2H, m		
4″	1.60, 1H, m 1.07, 1H, qt, *J* = 12.4, 3.3 Hz	26.43, CH_2_		1.60, 1H, m 1.07, 1H, qt, *J* = 12.4, 3.5 Hz	26.46, CH_2_

### Metabolite identification studies

Metabolite identification studies were carried out using the synthesised MT-45 and 2F-MT-45 reference standards. No previous metabolite identification studies have been carried out on MT-45 using human liver microsomes or hepatocytes, although metabolites have previously been identified using rat hepatocytes and detected in mouse urine [12]. MT-45 has been reported in biological samples from fatal and nonfatal intoxications of drug users; however, no metabolites have been identified in human samples. 2F-MT-45 has not previously been reported in any intoxication cases and thus this work gives information on urinary metabolites should such a case arise in the future or to provide metabolite data to be added to screening methods. Such studies are required to provide analytical targets for urinary metabolites for toxicological screening methods in clinical and forensic toxicology studies. At the physiological pH (7.4) at which all metabolomics studies were carried out, MT-45 and 2F-MT-45 are protonated and have log *D*_7.4_ values of 3.17 and 3.56 (see supplementary material), respectively, making them both relatively lipophilic substances.

**Fig. 5:**
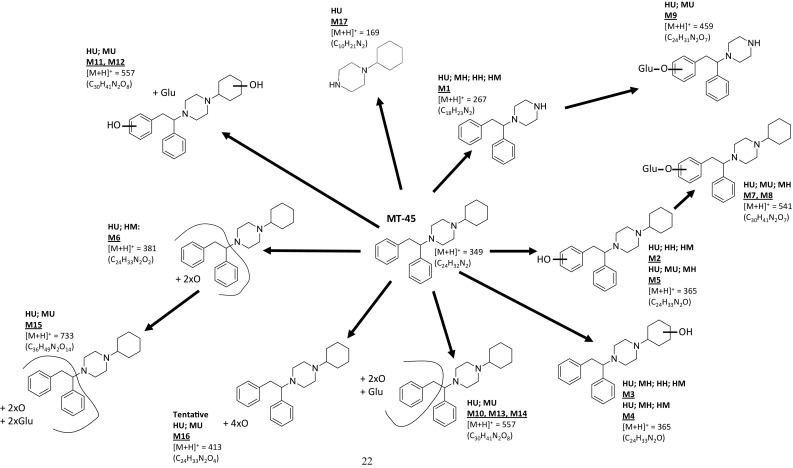
Overview of the metabolic profiling of MT-45 using human liver microsomes (HM), human hepatocytes (HH), mouse hepatocytes (MH), mouse urine (MU) from in vivo testing and authenticated human urine (HU) samples. Product ion spectra and structural elucidation data are provided in the supplementary material, section F

**Fig. 6:**
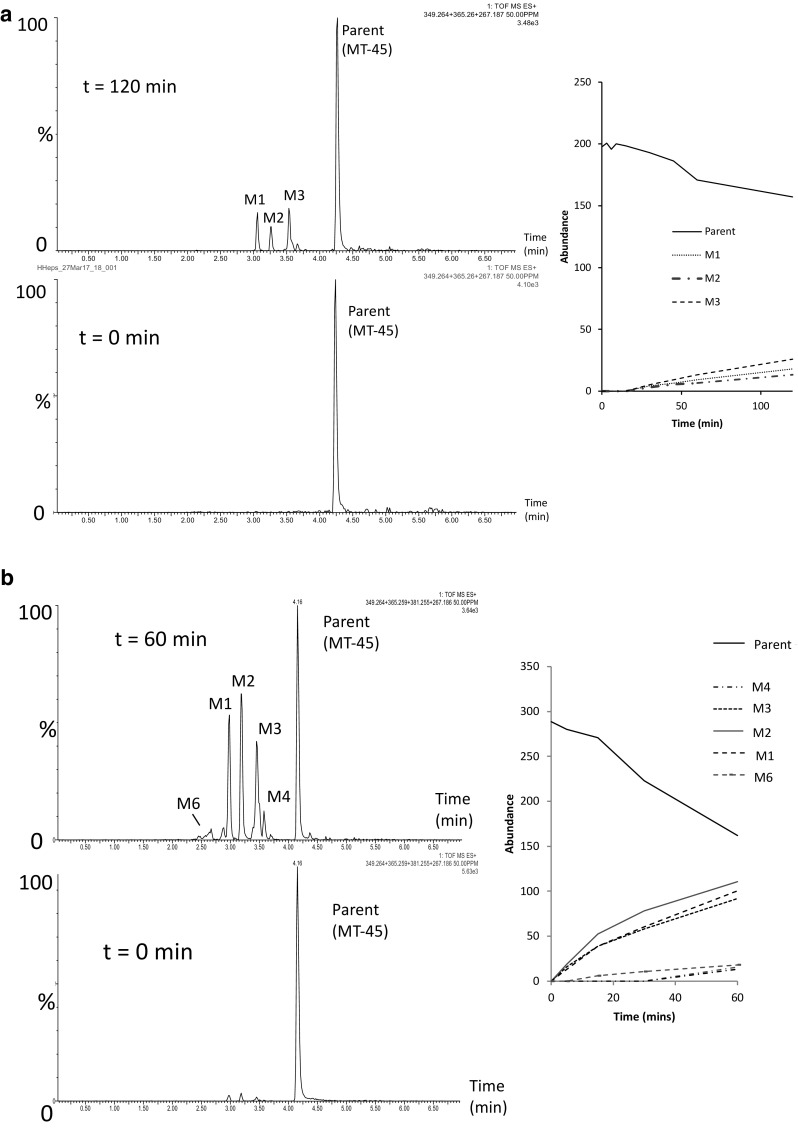
Chromatographic profiles and time-course data following incubation of MT-45 with **a** human hepatocytes after 120 min, and **b** human microsomes after 60 min obtained by ultra-performance liquid chromatography–quadrupole time-of-flight mass spectrometry. Metabolite labelling information can be found in Fig. 5 and Table 2

**Fig. 7:**
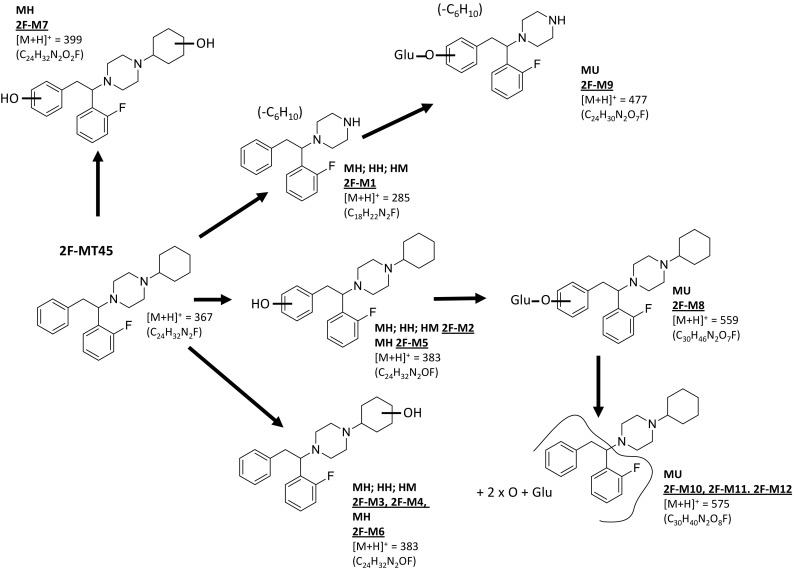
Overview of the metabolic profiling of 2F-MT-45 using human liver microsomes (HM), human hepatocytes (HH), mouse hepatocytes (MH) and mouse urine (MU) from in vivo testing. Product ion data and structural elucidation data are provided in supplementary material, section G

In a previous in vitro study using rat hepatocytes [12], the identification of 14 MT-45 metabolites (10 phase I and 4 phase II) was reported. In this study, 15 unique (plus 1, tentatively identified) MT-45 metabolites have been identified following MT-45 incubation with human liver microsomes, human hepatocytes, mouse hepatocytes and using mouse in vivo studies (see Fig. 5). An additional metabolite (M17), previously identified by [12], was identified in human urine only in this study. The chromatographic and mass spectral data for the metabolites identified (M1–M17) is provided in Table 2. Chromatograms and time-course data for MT-45 metabolite identification studies using human liver microsomes and human hepatocytes are provided in Fig. 6. Data for mouse hepatocyte and mouse in vivo experiments are provided in the supplementary information (section E). Twelve unique 2F-MT-45 metabolites were identified following 2F-MT-45 incubation with human liver microsomes, human hepatocytes, mouse hepatocytes and in vivo mouse studies, and these are summarised in Fig. 7 and Table 3 (2F-M1–2F-M12).

**Table 2:** Chromatographic and mass spectral data for MT-45 metabolites (phase I metabolites: M1–M6, M16 and M17; phase II metabolites: M7–M15)

Metabolite	Rt (min)	Measured mass^a^	Mol. formula [M + H]^+^	Calc. mass	Diff. (mDa)^b^	Diff. (ppm)^b^	Product ions (measured mass)^a^	Description
MT-45	4.25	349.2639	C_24_H_33_N_2_	349.2644	− 0.5	− 1.4	181.1024, 166.0795, 103.0566, 87.0934	Parent compound
M1	3.06	267.1881	C_18_H_23_N_2_	267.1861	2.0	7.5	181.1019, 166.0784, 103.0555, 87.0918	*N*-Dealkylation (–C_6_H_10_)
M2	3.26	365.2589	C_24_H_33_N_2_O	365.2593	− 0.4	− 1.1	197.0975, 169.1711, 119.0501, 87.0929	Mono-hydroxylation on phenethyl moiety
M3	3.53	365.2604	C_24_H_33_N_2_O	365.2593	1.1	3.0	185.1655, 181.0992, 166.0770, 103.0542, 87.0918	Mono-hydroxylation on cyclohexyl moiety
M4	3.68	365.2585	C_24_H_33_N_2_O	365.2593	− 0.8	− 2.2	185.1652, 181.1002, 166.0781, 103.0538, 87.0918	Mono-hydroxylation on cyclohexyl moiety
M5	3.47	365.2591	C_24_H_33_N_2_O	365.2593	− 0.2	− 0.5	197.0953, 169.1689, 119.0489, 87.0929	Mono-hydroxylation on phenethyl moiety
M6	2.76	381.2534	C_24_H_33_N_2_O_2_	381.2542	− 0.8	− 2.1	197.0970, 169.1727	Di-hydroxylation on diphenylethyl moiety
M7	2.73	541.2960	C_30_H_41_N_2_O_7_	541.2914	4.6	8.4	365.2771, 197.0970, 169.1730, 119.0542	Hydroxylation with subsequent glucuronidation on phenethyl moiety
M8	2.93	541.2918	C_30_H_41_N_2_O_7_	541.2914	4.0	7.3	365.2641, 197.0947, 169.1718, 119.0551	Hydroxylation with subsequent glucuronidation on phenethyl moiety
M9	1.64	459.2142	C_24_H_31_N_2_O_7_	459.2131	1.1	2.4	197.0977, 119.0498, 87.0935	*N*-Dealkylation (–C_6_H_10_) followed by hydroxylation and subsequent glucuronidation on phenethyl moiety
M10	2.18	557.2847	C_30_H_41_N_2_O_8_	557.2863	− 1.6	− 2.9	381.2525, 213.0910, 169.1701, 135.0437	Hydroxylation and hydroxylation with subsequent glucuronidation on phenethyl moiety
M11	2.32	557.2892	C_30_H_41_N_2_O_8_	557.2863	2.9	5.2	381.2511, 213.0899, 197.0942, 169.1701, 119.0502	Hydroxylation on the phenethyl moiety and hydroxylation on the cyclohexyl moiety and subsequent glucuronidation at one site
M12	2.41	557.2841	C_30_H_41_N_2_O_8_	557.2863	− 2.2	− 3.9	381.2534, 213.0901, 197.0976, 169.1678, 119.0488	Hydroxylation on the phenethyl moiety and hydroxylation on the cyclohexyl moiety and subsequent glucuronidation at one site
M13	2.64	557.2864	C_30_H_41_N_2_O_8_	557.2863	0.1	0.01	381.2534, 213.0909, 195.0797, 169.1707, 135.0443	Hydroxylation and hydroxylation with subsequent glucuronidation on phenethyl moiety
M14	2.70	557.2866	C_30_H_41_N_2_O_8_	557.2863	0.3	0.03	381.2523, 213.0905, 169.1703, 135.0447	Hydroxylation and hydroxylation with subsequent glucuronidation on phenethyl moiety
M15	2.31	733.3154	C_36_H_49_N_2_O_14_	733.3184	3.0	4.1	557.2855, 381.2471, 213.0903, 169.1699, 135.0453	Di-hydroxylation and subsequent glucuronidation on the diphenylethyl moiety
M16	2.98	413.2428	C_24_H_33_N_2_O_4_	413.2440	1.2	− 2.9	257.2023, 185.0598, 169.1696, 157.0640, 129.0710	4 × Hydroxylations at unknown positions Tentative identification
M17	2.07^c^	169.1704	C_10_H_21_N_2_	169.1705	0.1	− 0.3	87.0925, 83.0864	*N*-Dealkylation (–C_14_H_12_)

*Rt* retention time

^a^Where metabolites have been detected in multiple experiments, one measured mass is provided as an example

^b^Difference between measured mass and calculated mass for molecular formula predicted. Maximum permissible difference = 10 parts per million (ppm)

^c^Retention time using method detailed in [32, 33]

**Table 3:** Chromatographic and mass spectral data for 2F-MT-45 metabolites (phase I metabolites: 2F-M1–2F-M7; phase II metabolites: 2F-M8–2F-M12)

Metabolite	Rt (min)	Measured mass^a^	Mol. formula [M + H]^+^	Calc. mass	Diff. (mDa)^b^	Diff. (ppm)^b^	Product ions (measured mass)^a^	Description
2F-MT-45	4.54	367.2542	C_24_H_32_N_2_F	367.2550	− 0.8	− 2.2	181.1024, 166.0795, 103.0566, 87.0934	Parent compound
2F-M1	3.56	285.1782	C_18_H_22_N_2_F	285.1853	1.5	5.3	199.0935, 179.0660, 121.0463, 103.0548, 87.0928	*N*-Dealkylation (–C_6_H_10_)
2F-M2	3.61	383.2505	C_24_H_32_N_2_OF	383.2499	0.6		215.0671, 169.1721, 119.0499, 87.0938	Mono-hydroxylation on the phenethyl moiety
2F-M3	3.88	383.2500	C_24_H_32_N_2_OF	383.2499	0.2	0.4	199.0925, 179.0862, 121.0455	Mono-hydroxylation on the cyclohexyl moiety
2F-M4	4.01	383.2517	C_24_H_32_N_2_OF	383.2499	1.9	4.9	199.0925, 179.0862, 121.0455	Mono-hydroxylation on the cyclohexyl moiety
2F-M5	3.78	383.2501	C_24_H_32_N_2_OF	383.2499	0.3	0.7	215.0907, 169.1737, 119.0499, 87.0938	Mono-hydroxylation on the phenethyl moiety
2F-M6	4.13	383.2495	C_24_H_32_N_2_OF	383.2499	− 0.3	− 0.7	199.0919, 185.1661, 121.0455	Mono-hydroxylation on the cyclohexyl moiety
2F-M7	2.90	399.2441	C_24_H_32_N_2_O_2_F	399.2488	− 0.7	− 1.8	215.0913, 185.1647, 119.0524	Di-hydroxylation on the cyclohexyl and phenethyl moieties
2F-M8	3.16	559.2765	C_30_H_46_N_2_O_7_F	559.2820	− 5.5	− 9.8	215.0852, 119.0488, 87.0926	Hydroxylation with subsequent glucuronidation on the phenethyl moiety
2F-M9	1.95	477.2039	C_24_H_30_N_2_O_7_F	477.2037	0.2	0.4	215.0854, 119.0488, 87.0926	*N*-Dealkylation (–C_6_H_10_) followed by hydroxylation with subsequent glucuronidation on the phenethyl moiety
2F-M10	2.43	575.2762	C_30_H_40_N_2_O_8_F	575.2769	− 0.7	− 1.2	399.2435, 231.0826, 169.1708, 135.0448	Hydroxylation and hydroxylation with subsequent glucuronidation on the 2-fluorophenyl phenethyl moiety
2F-M11	2.87	575.2729	C_30_H_40_N_2_O_8_F	575.2769	− 4.0	− 7.0	399.2461, 231.0807, 169.1704	Hydroxylation and hydroxylation with subsequent glucuronidation on the 2-fluorophenyl phenethyl moiety
2F-M12	2.97	575.2726	C_30_H_40_N_2_O_8_F	575.2769	− 4.3	− 7.5	399.2433, 231.0811, 169.1712	Hydroxylation and hydroxylation with subsequent glucuronidation on the 2-fluorophenyl phenethyl moiety

*Rt* retention time

^a^Where metabolites have been detected in multiple experiments, one measured mass is provided as an example

^b^Difference between measured mass and calculated mass for molecular formula predicted. Maximum permissible difference = 10 parts per million (ppm)

#### Human in vitro studies

Five phase I MT-45 metabolites were detected following human in vitro studies (Fig. 6b). No phase II metabolites were detected in the human in vitro studies, suggesting that hepatic phase II metabolism was limited by the incubation conditions employed in this study. Three metabolites were identified following incubation of MT-45 with human hepatocytes: M1, formed as a result of *N*-dealkylation and M2 and M3, two mono-hydroxylated metabolites, (Figs. 5, 6a). Interestingly M1 (1,2-diphenylethylpiperazine) was not identified by Montesano et al. [12] in rat hepatocyte and mouse in vivo studies, who instead identified another *N*-dealkylated metabolite, 1-cyclohexyl-piperazine (labelled as M9 in that study, and M17 detected in human urine in this study).

M1 (1,2-diphenylethylpiperazine) was identified as one of three major metabolites, along with M2 and M3, using in vitro testing using human hepatocytes and human liver microsomes in this study and was also identified in verified human urine samples from users who had consumed MT-45 (Fig. 5). M1 is structurally similar to the dissociative substance, diphenidine (1,2-diphenylethylpiperadine; Fig. 1f). Two additional, minor (as estimated by peak area) metabolites were detected in the human liver microsome incubations only (Figs. 5 and 6b): the mono-hydroxylated metabolite M4 and the di-hydroxylated metabolite M6.

Four phase I 2F-MT-45 metabolites were detected following human in vitro studies (Fig. 7, Table 3); one metabolite resulted from *N*-dealkylation of the 2F-MT-45 parent structure and three due to mono-hydroxylations. As observed for MT-45, no phase II metabolites were detected in the human in vitro studies. Four metabolites were identified following incubation of 2F-MT-45 with human hepatocytes: 2F-M1 formed as a result of *N*-dealkylation and three mono-hydroxylated metabolites, 2F-M2, 2F-M3 and 2F-M4, were detected.

#### Mouse in vitro studies

Seven MT-45 metabolites were detected following incubation of MT-45 with mouse hepatocytes for 2 h (Fig. 5, Table 2; see supplementary material for chromatograms), three of which had not been observed previously in the human in vitro studies. M5 is hydroxylated on the phenethyl moiety similar to M2 detected in the human in vitro studies; M7 and M8 are phase II metabolites with glucuronidation occurring on the phenethyl moiety.

Seven 2F-MT-45 metabolites were detected following incubation of 2F-MT-45 with mouse hepatocytes (Fig. 7, Table 3), three of which (2F-M5, 2F-M6 and 2F-M7) had not been detected in the human in vitro studies. Metabolite 2F-M5, like 2F-M2, was hydroxylated on the phenethyl moiety; 2F-M6 is a minor metabolite which, like 2F-M3 and 2F-M4, was hydroxylated on the cyclohexyl moiety; 2F-M7 was dihydroxylated, with one hydroxylation on the phenethyl moiety and the other on the cyclohexyl moiety. The most prominent metabolites were the mono-hydroxylated 2F-M3 and 2F-M4 (*m*/*z* 383) metabolites.

#### Mouse in vivo studies

No MT-45 was detected in mouse urine collected after 24 h [see supplementary material, section E(ii)] and 10 metabolites were detected. Phase II (glucuronidated) metabolites were the most abundant as estimated by peak area, in agreement with previous findings in mouse urine [12]. Metabolite M9 was the glucuronidated form of the *N*-dealkylated metabolite M1. Metabolites M10–M14 contained one hydroxyl group and one hydroxyl group with subsequent glucuronidation. M11 and M12 were hydroxylated on both the cyclohexyl moiety and phenethyl moiety with one hydroxyl group being subsequently glucuronidated. M10, M13 and M14 were di-hydroxylated on the phenethyl moiety, as indicated by the product ion at *m*/*z* 135 with one hydroxyl group being subsequently glucuronidated. Metabolite M15 was di-hydroxylated and subsequently glucuronidated on the diphenylethyl moiety.

No 2F-MT-45 was detected in mouse urine collected after 24 h. Five glucuronidated metabolites were identified (Fig. 7, Table 3). Metabolite 2F-M8 was hydroxylated and subsequently glucuronidated on the phenethyl moiety. Metabolite 2F-M9 was the glucuronidated form of 2F-M1. Three other metabolites containing one hydroxyl group and one hydroxyl group with subsequent glucuronidation on the 2-fluorophenyl phenethyl moiety were detected: 2F-M10, 2F-M11 and 2F-M12.

#### Confirmatory identification of MT-45 metabolites in human urine

All MT-45 metabolites identified in the human and mouse in vitro and mouse in vivo studies (M1–M16) were detected in the human urine samples obtained from two analytically confirmed cases of MT-45 intoxication (Fig. 5), confirming the applicability of the methodology to case samples. The relative concentrations of metabolites were similar in both cases with M1 and M3 being the major metabolites, followed by M2 and M4 (each about 50% response in UPLC–QToF-MS as compared with M1 and M3), M5 and M6 (about 25%), M7–M9, M12 and M17 (~ 5–10%), and finally M10, M11, M13, M15 and M16 (~ 1% or less). An additional metabolite, M17, was detected in the human urine samples with similar peak areas to M5 and M6 (25–30% of M1 and M3). This *N*-dealkylated metabolite had not been identified in the human in vitro, the mouse in vitro and mouse in vivo studies using female C57 mice in this work, but had been previously identified as a significant metabolite in a study using rat hepatocytes and male ICR (CD-1^®^) mice [12].

## Discussion

2F-MT-45 was detected in a single seized tablet and its identity confirmed by comparison with in-house synthesised reference standards. It is noteworthy that there was some discrimination of the regioisomers using ATR-FTIR spectroscopy and Raman spectroscopy; however, they were most clearly distinguished using NMR spectroscopy, without the need to compare the resulting data with reference standards for structural elucidation. The sample was supplied in packaging labelled 2FPPP, indicating the presence of a dissociative drug with some structural similarities to 2F-MT-45. As far as the authors are aware, 2F-MT-45 has not been previously detected and the reasoning behind its synthesis in this case (deliberate or accidental) is unknown. This tablet was received for testing prior to the ban on production and export of MT-45 in China in July 2017 [29] and so it is possible that such compounds may enter the illicit drugs market in the future; however, MT-45 itself was relatively unpopular with users and a range of serious and unusual side effects have been linked with the drug [17, 20].

Both human hepatocyte and human liver microsomal incubations produced major *N*-dealkylated metabolites of MT-45 (M1) and 2F-MT-45 (2F-M1) which have structural similarities to dissociative drugs such as diphenidine and 2-FPPP. Although the same metabolites (M1 and 2F-M1) were observed after incubation with mouse hepatocytes, they were less prominent, suggesting that *N*-dealkylation is more efficiently expressed in the human in vitro systems. It is postulated that these metabolites, structurally related to diphenidine, will not be excreted directly via the kidney into urine but will instead be recirculated in plasma, and they are more likely to have a larger distribution volume in the body. It is hypothesised that these metabolites are pharmacologically active and will interact with NMDA receptors once recirculated, and that they may be responsible, at least in part, for the dissociative effects of MT-45 reported by some users. Further studies of the pharmacological activity of M1 (and 2F-M1) against opioid and NMDA receptors are required to test this hypothesis.

Cryopreserved primary human hepatocytes such as those used in this study provide a model in vitro metabolite identification solution. They incorporate natural hepatic metabolic enzyme systems, co-substrates for phase I and II metabolism at physiological levels, and intact drug transporter systems. They are theoretically most likely to provide an authentic range of human metabolites in metabolite identification studies [34, 35]. They are, however, expensive relative to other in vitro systems and are relatively more complex to use than microsomes, require incubation under carbogen and have lower intracellular glutathione (GSH) and glutathione-*S*-transferase (GST) activities than fresh hepatocytes. This means that, if occurring in vivo, GSH conjugation of MT-45 and 2F-MT45 may have been missed in vitro, although no evidence for GSH or GST conjugation was observed in the mouse in vivo study. In addition, human hepatocytes have a finite lifetime in suspension cultures (up to 5 h, although their activity is likely to decrease significantly after 2–3 h) and thus alternative in vitro systems are often used instead. Human liver microsomes represent a relevant system (and a more cost-effective approach) if the metabolism of the chemicals in question is known to be due to microsomal enzymes. In this study, the metabolites identified using human hepatocytes and human microsomes were very similar and three of the major MT-45 metabolites detected in human urine were also the major metabolites detected in the human in vitro methods. The use of pooled human S9 fraction or human liver microsomes combined with human cytosol has also been proposed as an alternative for NPS metabolomics studies focussed on identifying urinary target metabolites for toxicological screening purposes [36, 37].

The present study utilised a wide range of methodologies. MT-45 and 2F-MT-45 incubation with human hepatocytes produced relatively lower amounts of metabolites (and less metabolites) as compared to incubations with human liver microsomes (see Fig. 6 for MT-45 data). In both the human hepatocyte and human liver microsome experiments, only phase I metabolites were identified, suggesting that phase II metabolism of MT-45 and 2F-MT-45 is either very slow, or is extrahepatic, and these metabolites were not identified in the in vitro models used. Some minor phase II metabolism was observed in the mouse hepatocytes and these were also reported after MT-45 incubation with rat hepatocytes [12].

## Conclusions

A new fluorinated analogue of MT-45, 2F-MT-45, has been unequivocally identified in an illicitly produced tablet for the first time. The substance was identified using presumptive testing, GC–MS, UPLC–QToF-MS, ATR-FTIR spectroscopy, Raman spectroscopy and NMR spectroscopy in comparison to fully characterised in-house synthesised reference standards for MT-45 and 2F-, 3F- and 4F-MT-45. Metabolic profiling has been carried out for MT-45 and 2F-MT-45, and HRMS data for the identified metabolites has been reported in human in vitro testing for the first time. The presence of the identified MT-45 metabolites has been also confirmed in authenticated human urine samples. It is recommended that the protonated molecular ions of the parent and major metabolites (M1–M4 and 2F-M1–2F-M4) are added to toxicological screening methods, and that, where possible, HRMS data from previous analyses are re-examined using data-mining techniques to increase the toxicovigilance information available on the prevalence and use of such compounds in different jurisdictions.

## Electronic supplementary material

Below is the link to the electronic supplementary material.

Supplementary material 1 (DOCX 14187 kb)
