# Supplementary material for: Chemical synthesis, characterisation and in vitro and in vivo metabolism of the synthetic opioid MT-45 and its newly identified fluorinated analogue 2F-MT-45 with metabolite confirmation in urine samples from known drug users
Source: Forensic Toxicol. 2018 Apr 5;36(2):359–74. doi: 10.1007/s11419-018-0413-1 (PMC6002428; doi:10.1007/s11419-018-0413-1)
Supplement: Supplementary file 1 — Supplementary material 1 (DOCX 14187 kb) [file 11419_2018_413_MOESM1_ESM.docx]

**Chemical synthesis, characterisation and in vitro and in vivo metabolism of the synthetic opioid MT-45 and it’s newly identified fluorinated analogue 2F-MT-45 with metabolite confirmation in urine samples from known drug users**

Craig McKenzie^1^*, Oliver B Sutcliffe^2^*, Kevin D Read^3^, Paul Scullion^3^, Ola Epemolu^3^, Daniel Fletcher^3^, Anders Helander^4^, Olof Beck^4^, Alexia Rylski^4^, Lysbeth H. Antonides^1,2^, Jennifer Riley^3^, Shannah A Smith^1^, Niamh Nic Daeid^1^

* Corresponding authors

^1^ Forensic Drug Research Centre, Centre for Anatomy and Human Identification, School of Science and

Engineering, University of Dundee, Dundee, United Kingdom.

^2^ Division of Chemistry and Environmental Science, School of Science and the Environment, Manchester

Metropolitan University, Manchester, United Kingdom.

^3^ Drug Discovery Unit, School of Life Sciences, University of Dundee, Dundee, United Kingdom.

^4^ Department of Laboratory Medicine, Karolinska Institutet and Karolinska University Laboratory, Stockholm,

Sweden

**Supplementary Material**

**Section A Raman Spectroscopy Method Information**

***Raman Spectroscopy*** was performed using a proprietary analytical system developed at the University of Dundee. Powdered samples were prepared on #1 borosilicate cover slides (0.15mm thickness) therefore no sample dilution occurred. The system used 300 mW laser power on the back focal plane. A 780.24 nm laser was used to avoid fluorescence of any of the samples. Acquisition settings: 2s per spectrum, 35 accumulations. Any background fluorescence created was corrected using a spline function. Spectra were normalised to the highest peak. 100 x 1.25 NA oil immersion. Spectra were acquired using a 1200 lines/mm grid. Acquisition required multiple regions that were stitched together in post processing to produce a high spectral resolution (~0.06 cm^-1^). The 10 highest peaks were determined using a simple peak search algorithm and spectral shifts were corrected using a HgAr calibration light source.

**Section B *In vitro* Metabolite Identification Method Information**

***Pooled Human Liver Microsome (pHLM) incubations******:*** MT-45 and 2F-MT-45 and positive controls were incubated in HLM at 37^o^C with and without UDPGA. Verapamil was used as the positive control for incubations without UDPGA (phase I metabolism) and propranolol as the positive control for incubations with UDPGA (phase II metabolism). *Preparation of Cofactor Solutions:* 1 mmol/L solutions of NADPH (8.33 mg/mL) and 1 mmol/L UDPGA (6.46 mg/mL) in 50 mmol/L Potassium Phosphate Buffer (pH 7.4) were freshly prepared for every experiment and kept at 4°C. *Preparation of incubation mix:* Liver microsomes were thawed rapidly in a water bath at 37ᵒC and kept on ice until use. They were diluted with 50 mmol/L potassium phosphate buffer pH 7.4 to obtain the protein concentration of 1.11 mg/mL. *Preparation of test compounds and positive controls:* 10 mmol/L stock solutions of MT-45 and 2F-MT-45 and positive control (propranolol or Verapamil) were prepared in DMSO and 500 μmol/L working solutions were prepared in 50:50 acetonitrile:water. *Procedure:* 450 µL of liver microsomes (1.11 mg/mL) and 45 µL of cofactor (UDPGA or UDPGA/NADPH) were added to wells of a 96 deep-well 2 mL plate (incubation plate) kept at 37ᵒC. 5 µL of each test compound was added and a 50 µL aliquot of the incubation mixture was immediately removed and added to 200 µL of acetonitrile to terminate the reaction, providing an initial (t=0) sample. Further 50 µL aliquots of the incubation mixtures were removed at 5, 15, 30 and 60 min. All samples were centrifuged to sediment any precipitated protein (3270 rpm for 10 min). 150 µL of the supernatant was removed and diluted with 50 µL milli-Q water in a 96 deep-well 2 mL plate (analysis plate) and sealed prior to analysis by UPLC-QToF. ***Human and Mouse Hepatocyte Incubations:*** *In vitro* metabolic stability studies were performed using cryopreserved hepatocytes; either mouse (Mheps - Mouse CD1 Cryo Hep Female Suspension Pool 20 Donor, 4-8 million recoverable cells, MSCS20, Life Technologies Ltd, UK) or human (Hheps - Human Cryo Hep Suspension Pool 50 Donor, mixed gender, HMCS50, Life Technologies Ltd, UK). MT-45 and 2F-MT-45 were incubated in Mheps and Hheps. 7-Ethoxycoumarin (7-EC) and (7-HC) were used as positive controls. *Reagents:* Williams Media E (WME), Without Glutamine or Phenol Red (A12176-01); Cell Maintenance Supplement Pack including Cocktail B/Dexamethasone (CM4000); Trypan Blue (15250-061), CHRM cell recovery media (CM7000), all purchased from Gibco/Invitrogen Life Technologies. Acetonitrile and Methanol were HPLC grade and water was Milli-Q. *Preparation of supplemented media for hepatocytes:* the entire volume of the cell maintenance supplement pack was added to 500 mL of WME and mixed and stored at 37^o^C for > 30 min prior to use. *Procedure:* *Preparation of Cryopreserved Hepatocytes*: Vials of cryopreserved hepatocytes were thawed and the ice pellet was re-suspended in 50 mL of cell resuspension media (CHRM) and gently mixed. The hepatocyte solution was centrifuged at 55 × g for 3 min for mouse hepatocytes and 100 x g for 10 mins for human hepatocytes at room temperature. The supernatant was removed and the cell pellet gently re-suspended in supplemented WME to a final volume of 1 mL. A 10 μL aliquot was transferred to a micro tube containing 10 μL of trypan blue solution and 80 μL of supplemented WME providing a 1 in 10 dilution of cells. The solution was gently mixed and an aliquot was placed onto a haemocytometer for counting. Supplemented WME was further added to the cell suspension to give a final cell concentration of 1.4 × 10^6^ cells/mL. 200 μL of cell suspension was added to the appropriate wells of a 48 well cell culture plate and incubated at 37^o^C with gentle agitation for approximately 10 min. *Preparation of Test Compounds in Cell Media:* 100 μmol/L stock solutions of MT-45 and 2F-MT-45 and the positive control, 7-EC were prepared in DMSO and 1 mL of 5 μmol/L working solution (final DMSO concentration 1%) was prepared in supplemented WME in a 48-well cell culture plate. *Reaction initiation and sampling:* 200 μL of working solution containing test compound was added to 200 μL of cell suspension to initiate the reaction. A 20 μL aliquot of the incubation mixture was removed immediately and added to 80 μL of acetonitrile containing an internal standard (50 ng/mL donepezil). Further 20 aliquots were removed to a 96 deep-well 2 mL plate (analysis plate) at the following timepoints: 3, 6, 9, 15, 30, 60, 90 and 120 min. A ‘no hepatocyte’ control containing 200 μL of test compounds in supplemented WME and 200 μL of supplemented WME was prepared for each test compound to measure compound stability over the course of the experiment. 20 μL aliquots were taken at 0 and 120 min. 100 μL of 80:20 water:acetonitrile was added to all samples and the analysis plate was centrifuged at 2800 rpm for 10 min at room temperature prior to injection and analysis of samples by UPLC-QToF.

**Section C Reference standard characterization data**

1. **Presumptive colour test results for MT-45 and it’s fluorinated analogues**

| **Test/Compound** | **MT-45** | **2F-MT-45** | **3F-MT-45** | **4F-MT-45** |
| --- | --- | --- | --- | --- |
| **Marquis** | Immediate: yellow  After 5 mins: Orange/Brown | Immediate: yellow  After 5 mins: Orange/Green | Immediate: yellow  After 5 mins: Orange/Brown | Immediate: yellow  After 5 mins: Orange/Green |
| **Mecke** | Immediate: faint yellow  After 5 mins: Yellow/green | Immediate: faint yellow  After 5 mins: Yellow/green | Immediate: faint yellow  After 5 mins: Yellow/green | Immediate: faint yellow  After 5 mins: Yellow/green |
| **Scott’s** | Immediate: blue  After 5 mins: blue | Immediate: blue  After 5 mins: blue | Immediate: blue  After 5 mins: blue | Immediate: blue  After 5 mins: blue |
| **Modified Scott’s** | Step1: +ve (blue)  Step 2: -ve (blue)  Step 3: -ve  (no blue/pink layers) | Step1: +ve (blue)  Step 2: -ve (blue)  Step 3: -ve  (no blue/pink layers) | Step1: +ve (blue)  Step 2: -ve (blue)  Step 3: -ve  (no blue/pink layers) | Step1: +ve (blue)  Step 2: -ve (blue)  Step 3: -ve  (no blue/pink layers) |
| **Liebermann** | Immediate: red/brown  After 5 mins: dark red/brown | Immediate: orange  After 5 mins: orange | Immediate: red/brown  After 5 mins: dark red/brown | Immediate: red/brown  After 5 mins: dark red/brown |
| **Mandelin** | Immediate: brown  After 5 mins: green | Immediate: brown  After 5 mins: green | Immediate: brown  After 5 mins: green | Immediate: brown  After 5 mins: green |
| **Froehde** | Immediate:  light brown  After 5 mins: light brown | Immediate:  light brown  After 5 mins: light brown | Immediate:  light brown  After 5 mins: light brown | Immediate:  light brown  After 5 mins: light brown |

1. Experimentally derived physico-chemical parameters for MT-45 and it’s fluorinated analogues

| **Compound** | **pKa_1_** | **pKa_2_** | **LogP** | **LogD_7.4_** |
| --- | --- | --- | --- | --- |
| MT-45 | 3.37 (3.36) | 9.23 (8.17) | 4.01 | 3.17 |
| 2F-MT-45 | 2.42 (2.89) | 9.81 (8.12) | 4.35 | 3.56 |
| 3F-MT-45 | 3.30 (3.04) | 8.45 (8.15) | 4.60 | 3.78 |
| 4F-MT-45 | 3.20 (3.28) | 9.42 (8.17) | 4.42 | 3.59 |

1. **NMR**

**MT-45**

**¹H NMR:** (500 MHz, 25°C, CDCl_3_) δ ^1^H (ppm) = 7.22 - 7.19 (2H, m, ArCH), 7.17 - 7.14 (1H, m, ArCH),
7.13 - 7.09 (4H, 2m, ArCH), 7.09 - 7.05 (1H, m, ArCH), 6.95 - 6.93 (2H, m, ArCH), 3.52
(1H, dd, *J* = 9.7, 5.0 Hz, **CH**CH_2_), 3.33 (1H, dd, *J* = 13.3, 5.0 Hz, CH**CH_2_**), 2.94 (1H, dd, *J* = 13.3, 9.7 Hz, CH**CH_2_**), 2.7 - 2.4 (8H, 2br, N(CH_2_CH_2_)_2_N), 2.17 (1H, tt, *J* = 10.8, 3.3 Hz, NCH), 1.86 (2H, brd), 1.77 (2H, brd), 1.61 (1H, brd), 1.27 - 1.03 (5H, 3m)

**^13^C NMR:** (126 MHz, 25°C, CDCl_3_) δ ^13^C (ppm) = 139.7 (s, ArC), 139.7 (s, ArC), 129.5 (s, ArCH), 129.0 (s, ArCH), 128.0 (s, ArCH), 128.0 (s, ArCH), 127.1 (s, ArCH), 125.8 (s, ArCH), 72.3 (s, **CH**CH_2_), 63.6 (s, NCH), 51.1 (br, N**CH_2_**CH_2_N), 49.4 (s, NCH_2_**CH_2_**N), 39.5 (s, CH**CH_2_**), 29.2 (s, CH2), 29.1 (s, CH2), 26.4 (s, CH2), 26.0 (s, CH2)

**2F-MT45**

**^1^H-NMR:** (500 MHz, 25^o^C, CDCl_3_) δ 7.29 (1H, td, *J* = 7.4, 1.8 Hz, ArCH), 7.16 – 7.04 (7H, 5 m, ArCH), 6.88 (1H, ddd, *J* = 10.2, 8.2, 1.2 Hz, ArCH), 4.12 (1H, dd, *J* = 9.7, 5.6 Hz, **CH**CH_2_), 3.31 (1H, dd, *J* = 13.5, 5.6 Hz, CH**CH_2_**), 3.04 (1H, dd, *J* = 13.5, 9.7 Hz, CH**CH_2_**), 2.7 – 2.4 (8H, 2br, N(CH_2_CH_2_)_2_N), 2.17 (1H, tt, *J* = 10.9, 3.3 Hz, NCH), 1.86 (2H, brd), 1.77 (2H, brd), 1.60 (1H, brd), 1.25 – 1.03 (5H, 3m)

**^13^C-NMR:** (126 MHz, 25^o^C, CDCl_3_) δ 161.5 (d, *J* = 244.8 Hz, ArCF), 139.4 (s, ArC), 130.1 (d, *J* = 4.9 Hz, ArCH), 129.4 (s, ArCH), 128.6 (d, *J* = 8.5 Hz, ArCH), 128.1 (s, ArCH), 126.0 (s, ArCH), 125.7 (d, *J* = 14.7 Hz, ArC), 123.6 (d, *J* = 3.3 Hz, ArCH), 115.4 (d, *J* = 23.8 Hz, ArCH), 63.6 (s, NCH or **CH**CH_2_), 63.5 (s, **CH**CH_2_ or NCH), 50.4 (br, N**CH_2_**CH_2_N), 49.4 (s, NCH_2_**CH_2_**N), 38.4 (d, *J* = 1.2 Hz, CH**CH_2_**), 29.2 (s, CH2), 29.2 (s, CH2), 26.5 (s, CH2), 26.0 (s, CH2)

**^19^F-NMR:** δ -119.6

**3F-MT45**

**^1^H-NMR:** (500 MHz, 25^o^C, CDCl_3_) δ 7.16 – 7.07 (4H, 3m, ArCH), 6.96 – 6.92 (2H, m, ArCH), 6.89 – 6.82 (3H, 3m, ArCH), 3.52 (1H, dd, *J* = 9.7, 5.0 Hz, **CH**CH_2_), 3.31 (1H, dd, *J* = 13.3, 5.0 Hz, CH**CH_2_**), 2.88 (1H, dd, *J* = 13.3, 9.7 Hz, CH**CH_2_**), 2.75 – 2.35 (8H, 2br, N(CH_2_CH_2_)_2_N), 2.18 (1H, tt, *J* = 10.9, 3.2 Hz, NCH), 1.87 (2H, brd), 1.77 (2H, brd), 1.61 (1H, m), 1.27 – 1.04 (5H, 3m)

**^13^C-NMR:** (126 MHz, 25^o^C, CDCl_3_) δ 162.8 (d, *J* = 245.1 Hz, ArCF), 142.7 (d, *J* = 6.5 Hz, ArC), 139.2 (s, ArC), 129.5 (s, ArCH), 129.3 (d, *J* = 8.2 Hz, ArCH), 128.1 (s, ArCH), 126.0 (s, ArCH), 124.7 (d, *J* = 2.7 Hz, ArCH), 115.5 (d, *J* = 21.1 Hz, ArCH), 113.9 (d, *J* = 21.2 Hz, ArCH), 71.8 (d, *J* = 1.5 Hz, **CH**CH_2_), 63.6 (s, NCH), 51.1 (br, N**CH_2_**CH_2_N), 49.4 (s, NCH_2_**CH_2_**N), 39.5 (s, CH**CH_2_**), 29.2 (s, CH2), 29.1 (s, CH2), 26.4 (s, CH2), 26.0 (s, CH2)

**^19^F-NMR:** δ -117.2

**4F-MT45**

**^1^H-NMR:** (500 MHz, 25^o^C, CDCl_3_) δ 7.14 – 7.03 (5H, 3m, ArCH), 6.92 – 6.90 (2H, m, ArCH), 6.90 – 6.86 (2H, m, *J* = 8.7 Hz, ArCH), 3.49 (1H, dd, *J* = 10.0, 4.9 Hz, **CH**CH_2_), 3.32 (1H, dd, *J* = 13.2, 4.9 Hz, CH**CH_2_**), 2.86 (1H, dd, *J* = 13.2, 10.0 Hz, CH**CH_2_**), 2.80 – 2.30 (8H, 2 br, N(CH_2_CH_2_)_2_N), 2.18 (1H, tt, *J* = 10.9, 3.3 Hz), 1.87 (2H, brd), 1.77 (2H, brd), 1.61 (1H, brd), 1.27 – 1.04 (5H, 3m)

**^13^C-NMR:** (126 MHz, 25^o^C, CDCl_3_) δ 162.0 (d, *J* = 244.7 Hz, ArCF), 139.3 (s, ArC), 135.6 (d, *J* = 3.2 Hz, ArC), 130.3 (d, *J* = 7.8 Hz, ArCH), 129.5 (s, ArCH), 128.1 (s, ArCH), 125.9 (s, ArCH), 114.7 (d, *J* = 21.1 Hz, ArCH), 71.6 (s, **CH**CH_2_), 63.6 (s, NCH), 51.1 (br, N**CH_2_**CH_2_N), 49.4 (s, NCH_2_**CH_2_**N), 39.7 (s, CH**CH_2_**), 29.2 (s, CH2), 29.2 (s, CH2), 26.5 (s, CH2), 26.0 (s, CH2)

**^19^F-NMR:** δ -119.2

1. **GC-MS (Electron Impact (EI) Ionisation Mode)**

**MT-45**

Principal ions at *m/z* 257, 258, 91, 107, 55, 56, 165, 103, 166; M^+^ at *m/z* = 348

**
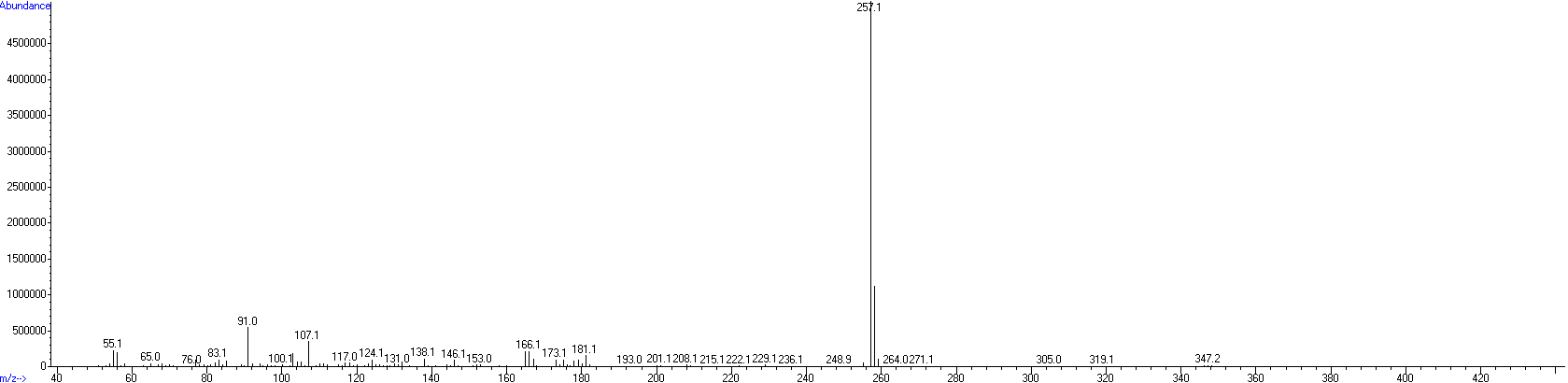
**

**2F-MT-45**

Principal ions at m/z 275, 276, 109, 116, 55,56, 103, 138, 91, 164; M^+^ at *m/z* = 366

**
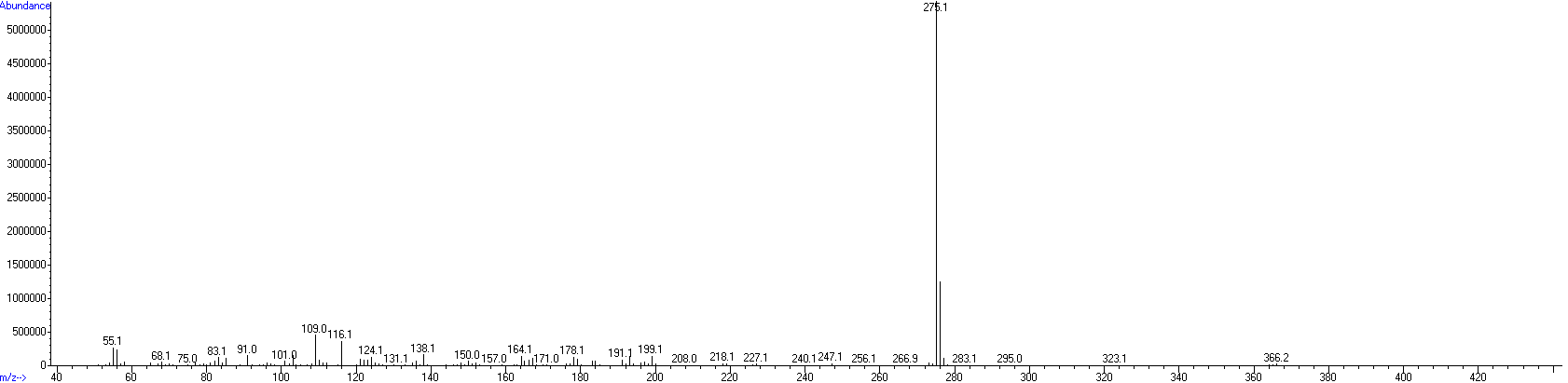
**

**3F-MT-45**

Principal ions at m/z 275, 276, 109, 116, 55,56, 103, 138, 91, 164; M^+^ at *m/z* = 366

**
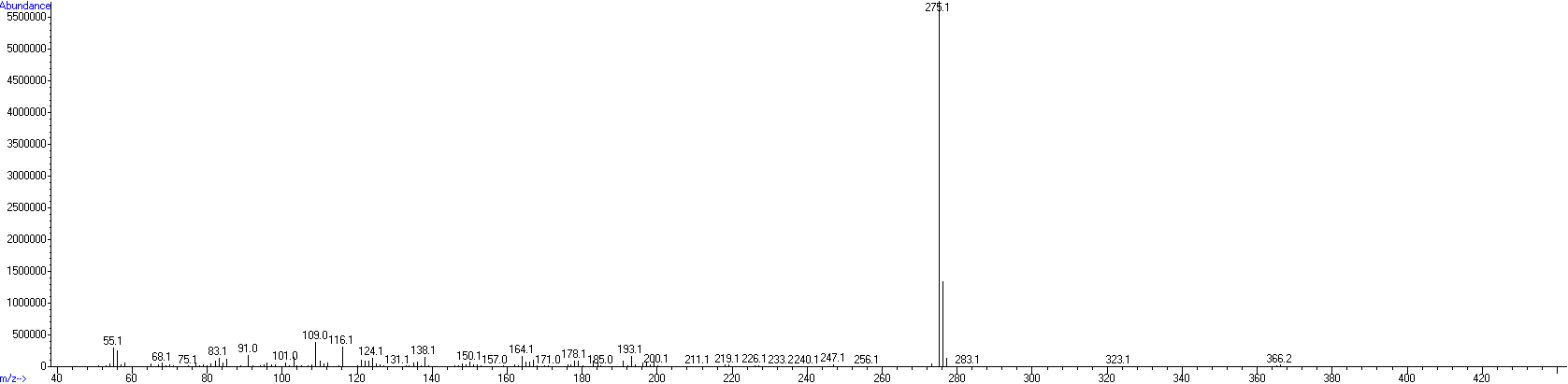
**

**4F-MT-45**

Principal ions at m/z 275, 276, 109, 116, 55,56, 103, 138, 91, 164; M^+^ at *m/z* = 366

**
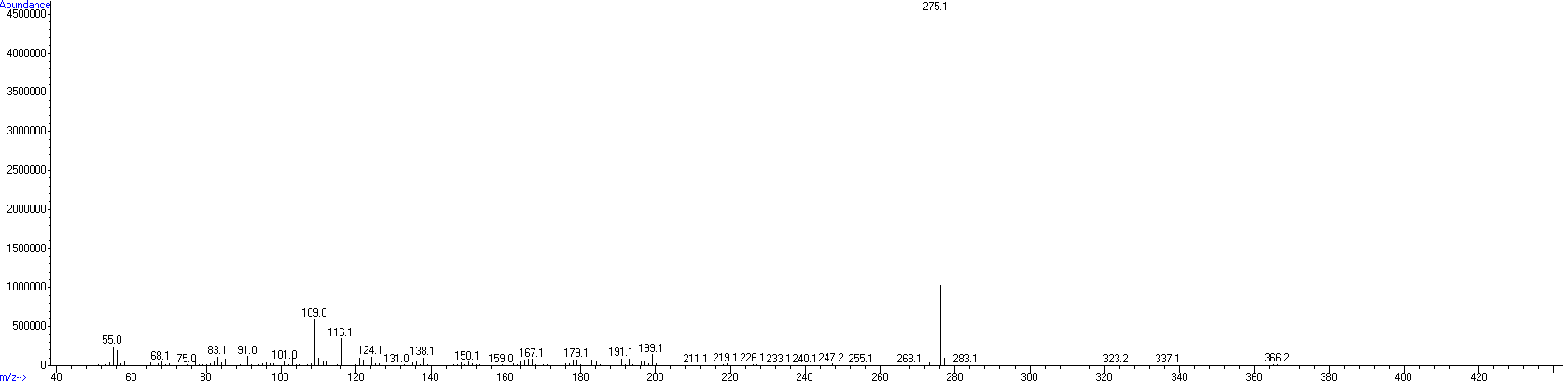
**

1. **GC-MS analysis of (a) MT-45, fluorinated MT-45 analogues reference standards and (b) the seized tablet from bag labelled ‘2FPPP’.**

1. **UPLC-QToF MS/MS Spectra**

**MT-45**

**
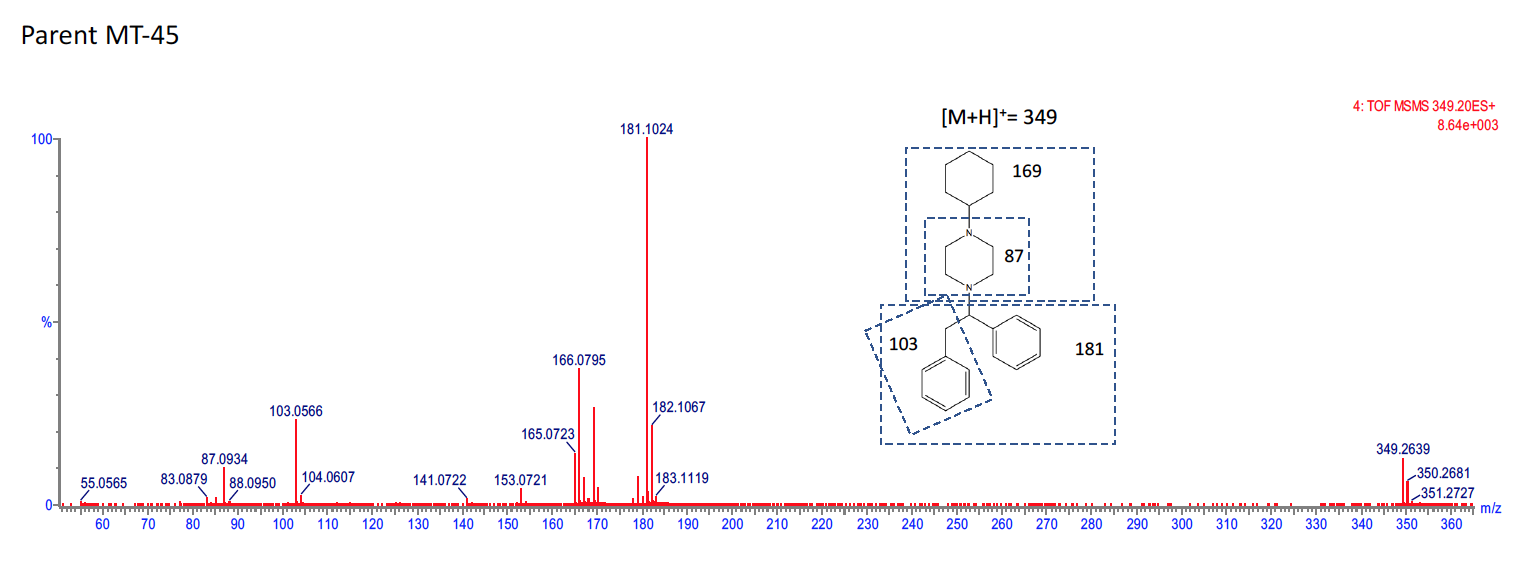
**

**2F-MT-45**

**
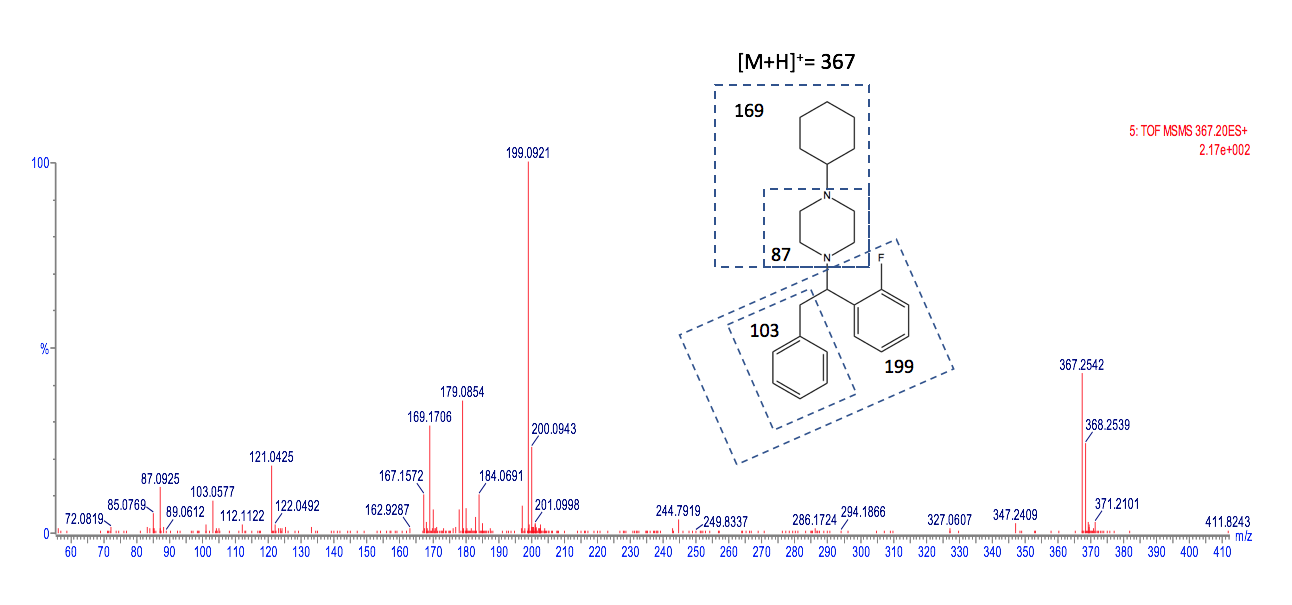
**

**3F-MT-45**


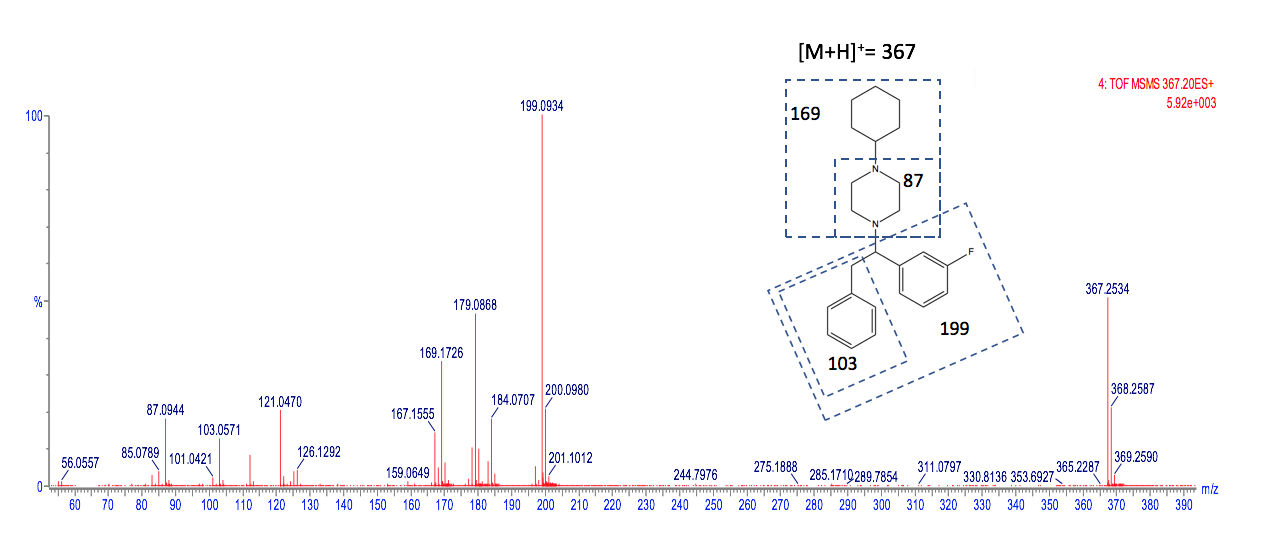


**4F-MT-45**


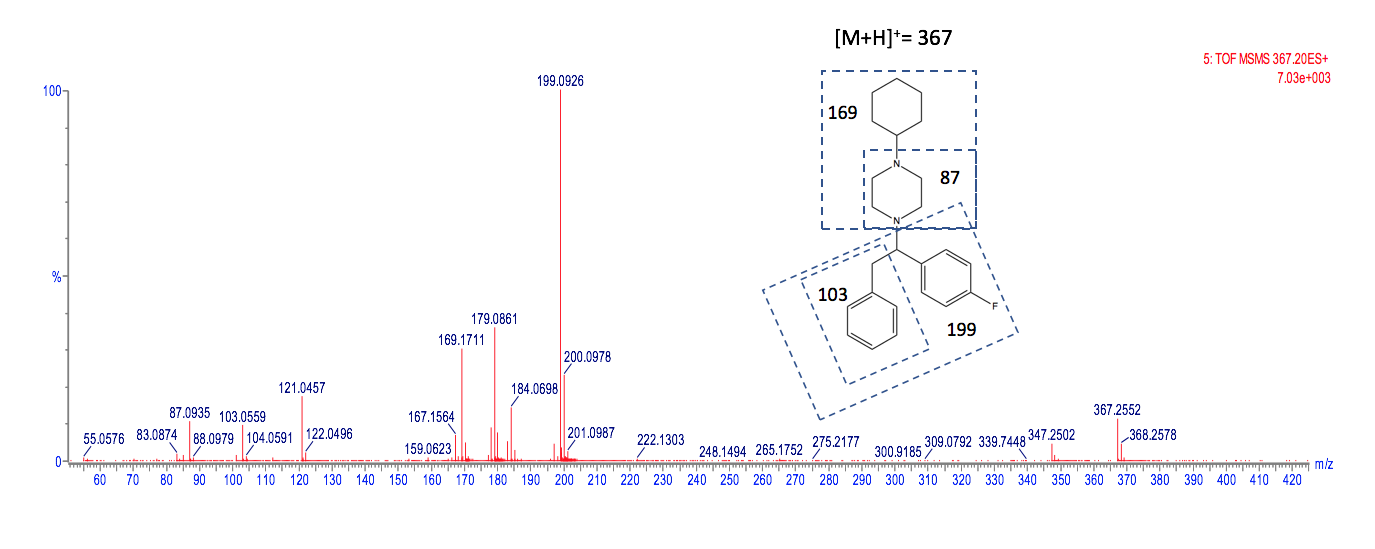


1. **ATR-FTIR analysis of MT-45 and fluorinated MT-45 analogue reference standards**

**and the seized tablet from bag labelled ‘2FPPP’.**

1. **Raman Spectroscopy**

**MT-45**

Principal peaks at wavenumbers 1003, 781, 1210, 469, 1036, 497, 619, 481, 437, 759 cm^-1^

**2F-MT-45**

**Raman:** Principal peaks at wavenumbers: 999, 850, 1030, 1231, 481, 525, 1445, 1155, 742, 1058

**3F-MT-45**

**Raman** Principal peaks at wavenumbers: 1000, 468, 844, 1444, 1040, 710, 517, 490, 1027, 528

**4F-MT-45**

**Raman** Principal peaks at wavenumbers: 1000, 828, 848, 777, 1203, 469, 1444, 1040, 1598, 1027

**Section D Seized Sample 2D-NMR Characterisation Data and Raman spectroscopy**

**comparison**

1. COSY-DQF Spectra


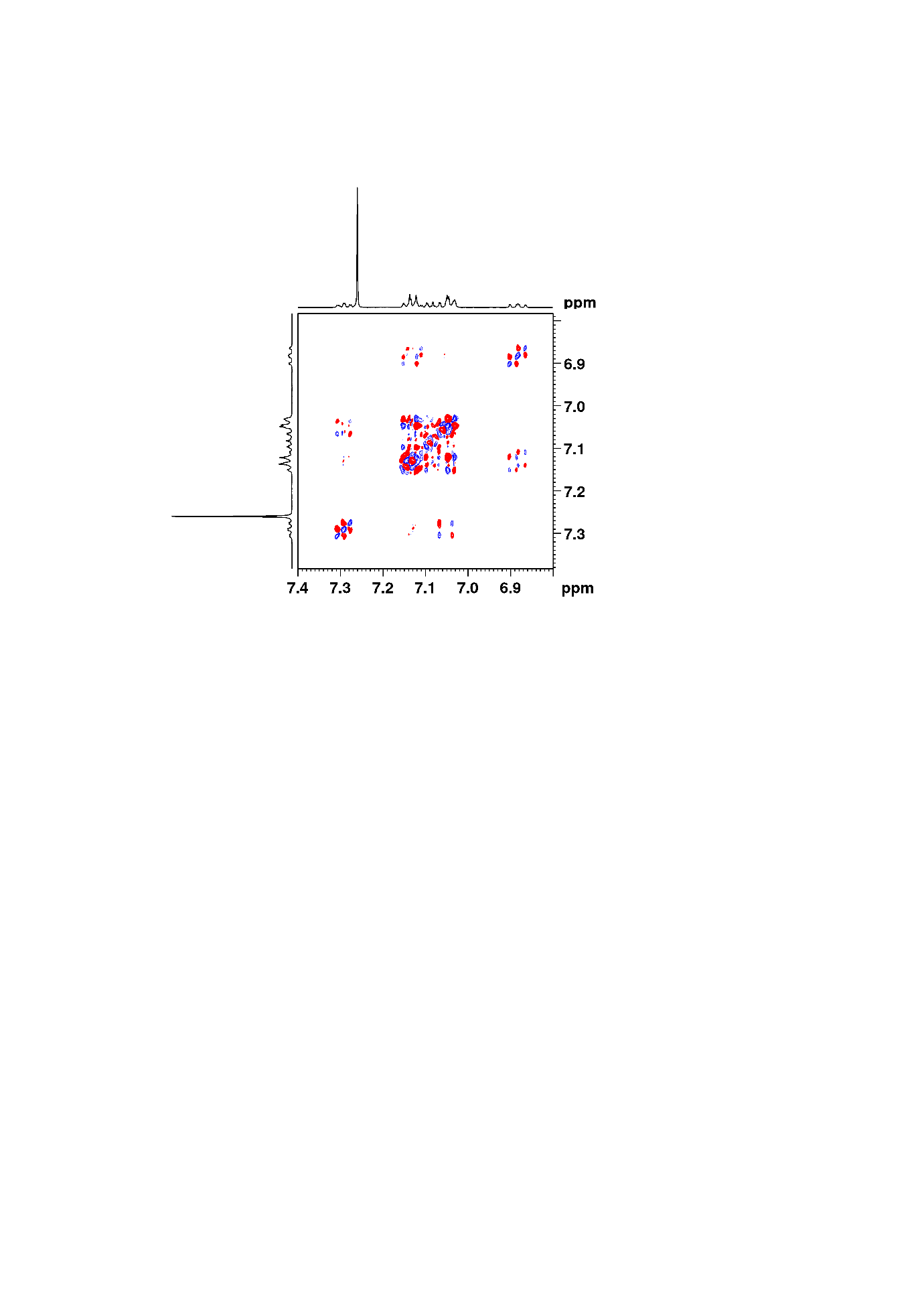

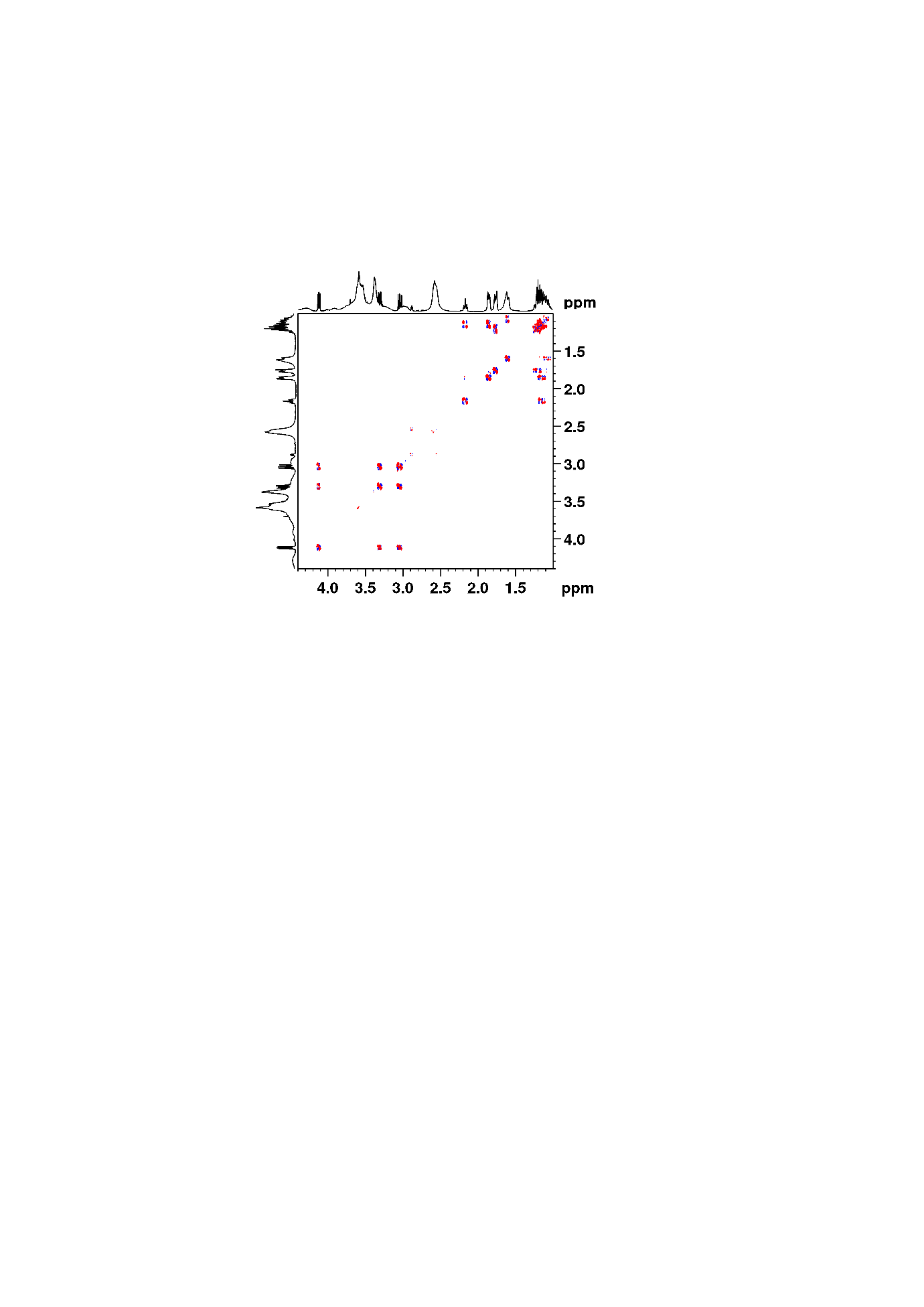


1. Overview of HSQC Spectra (red CH, Blue CH_2_)


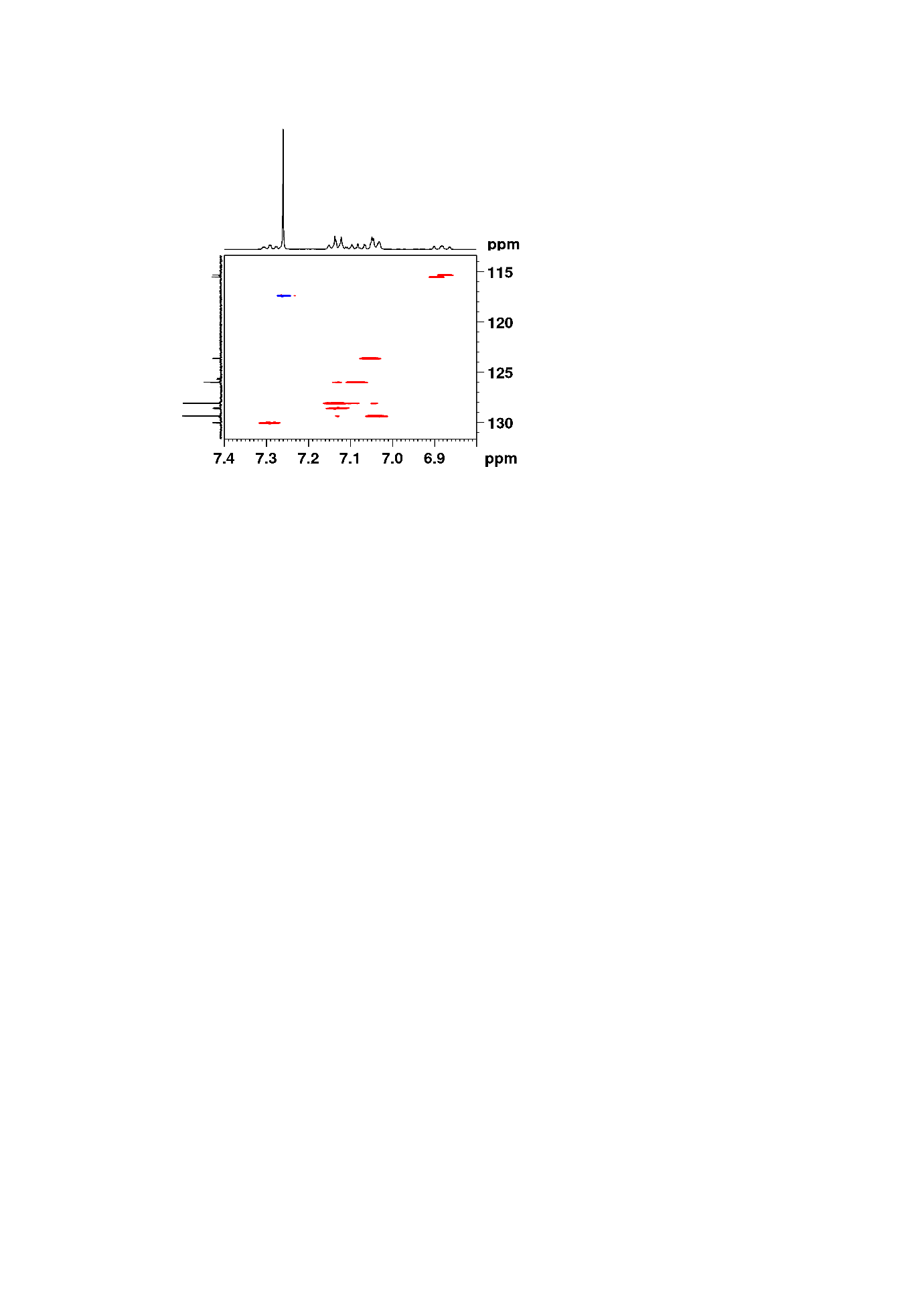

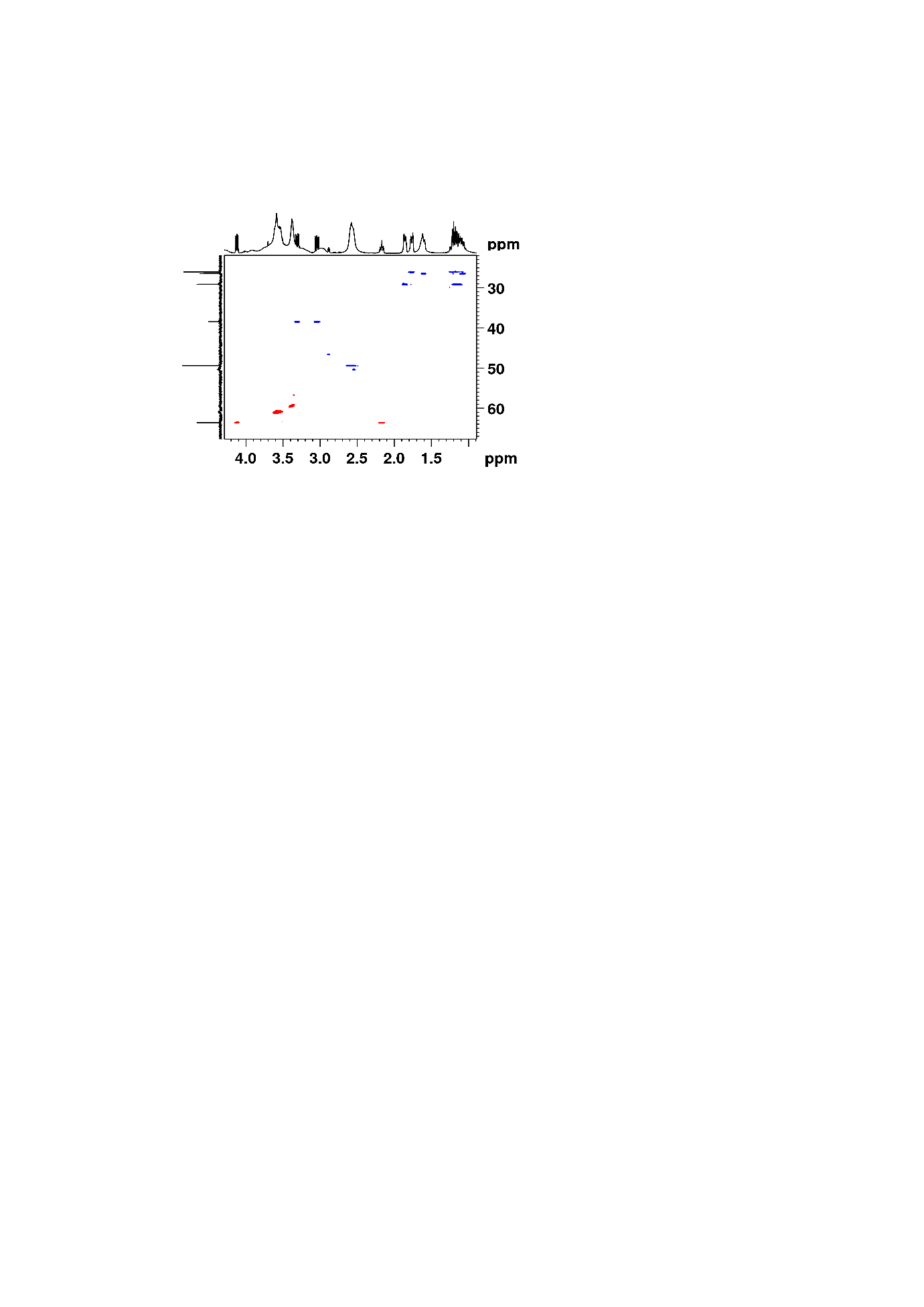


CHCl_3_
aliased

1. Overview of HMBC Spectrum


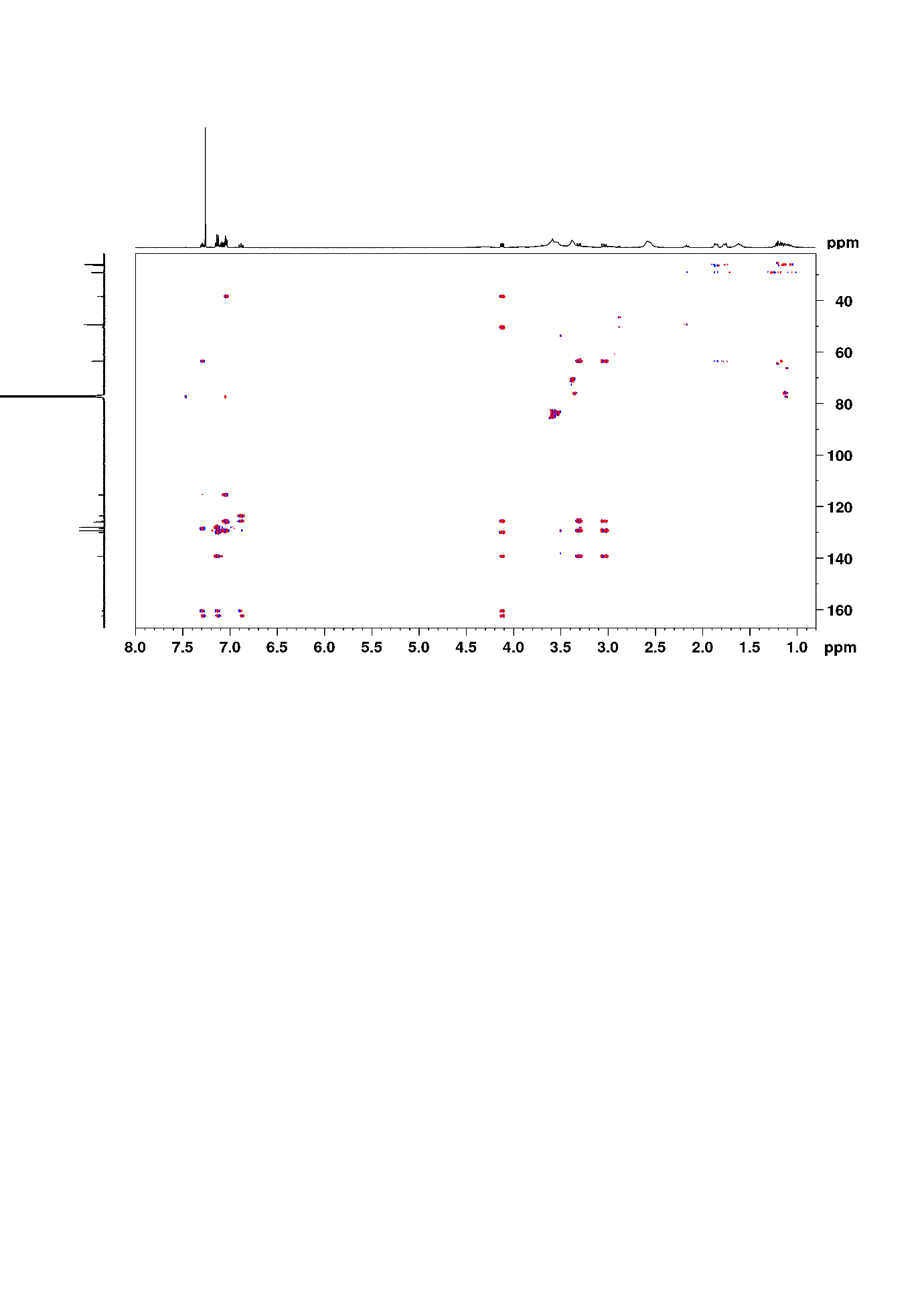


1. ^13^C Spectrum


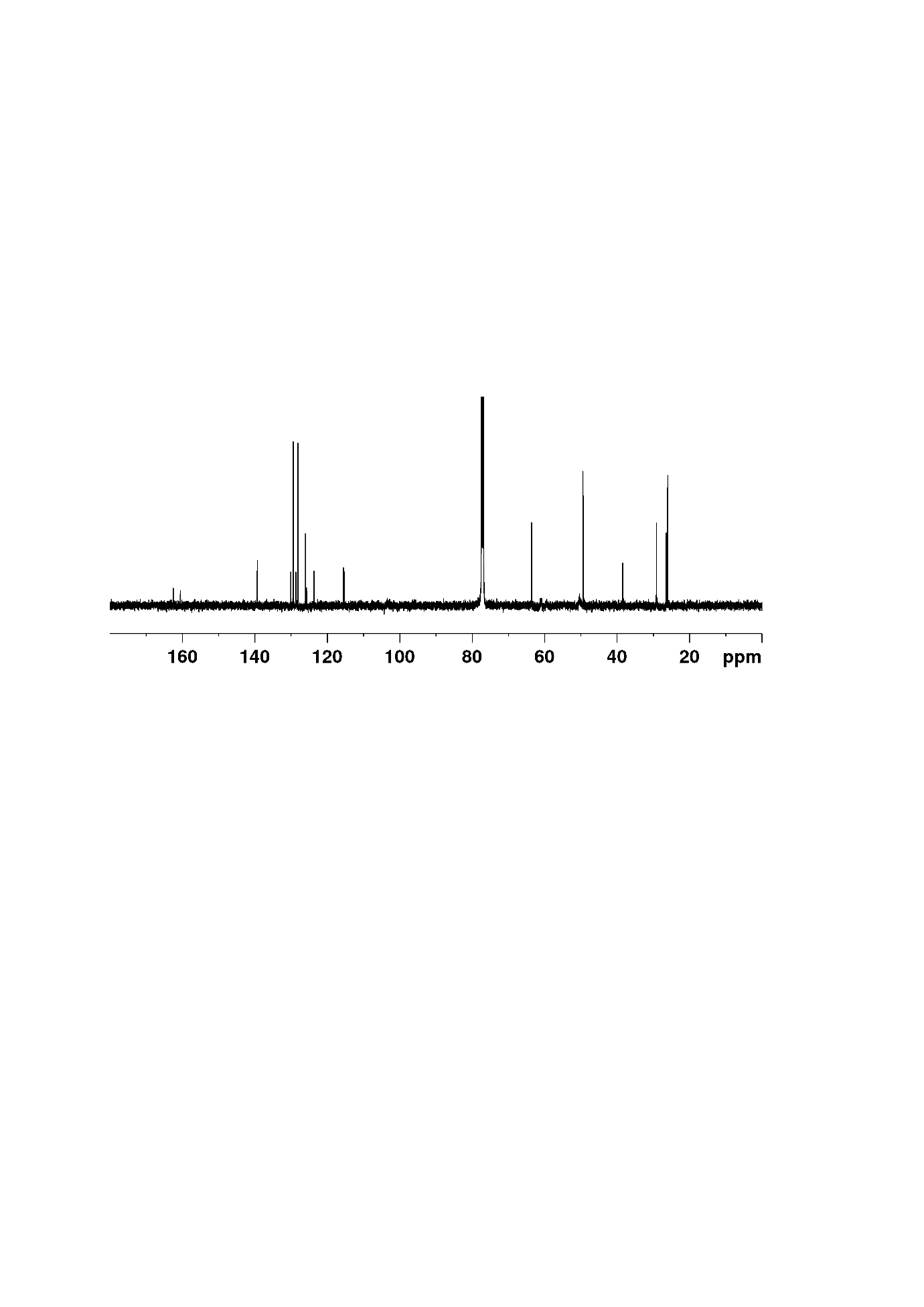


1. Labelled HSQC spectra for the seized sample


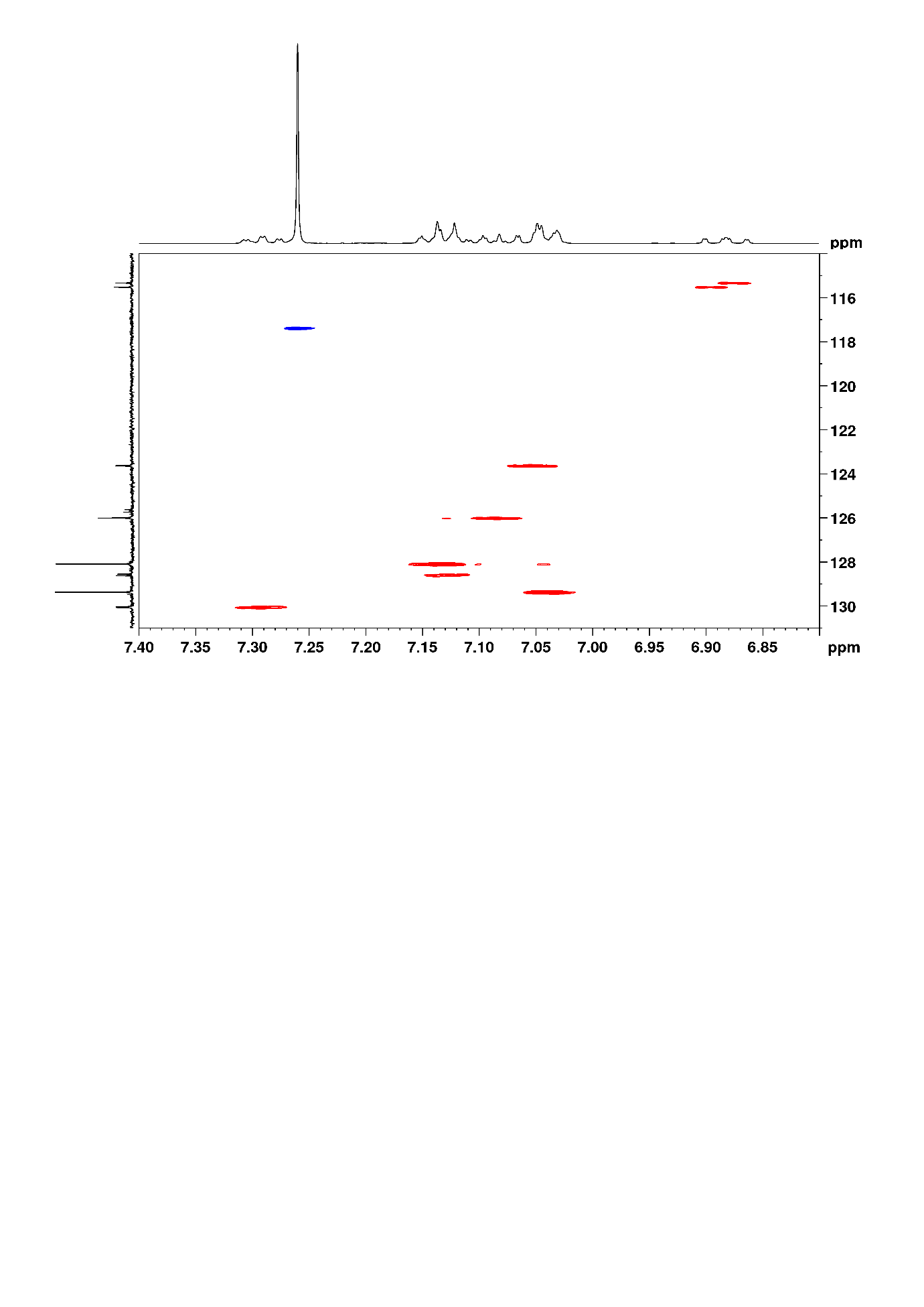


1’-3

2’-2,6

1’-5

2’-4

1’-4

2’-3,5

1’-6

CHCl_3_
aliased


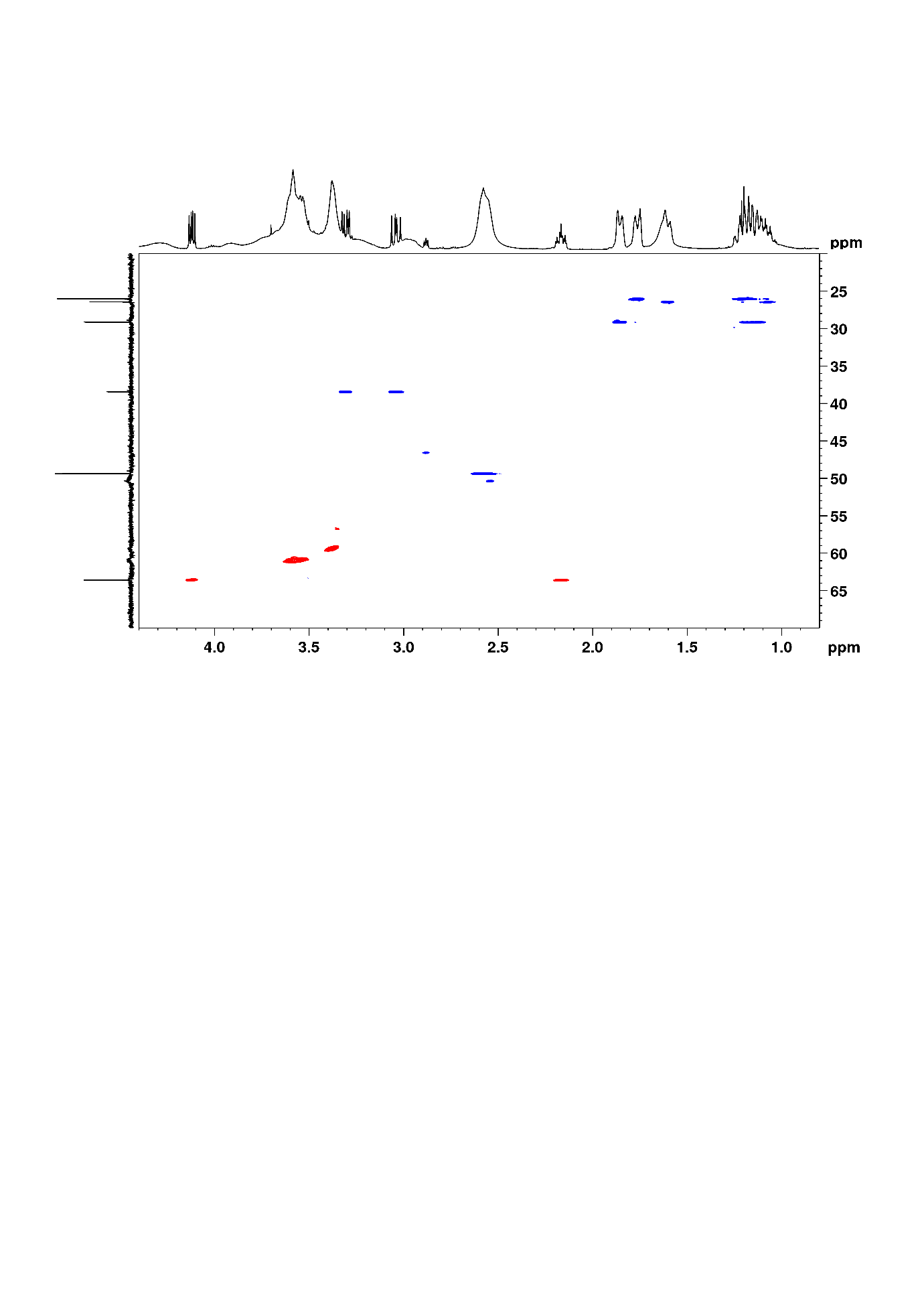


Excipient

4’’

2’’,6’’

3’’,5’’

1’’

2,6

3,5

1’

2’

1. Expansions of the HMBC spectrum of the seized sample ^1^H{^13^C}
2. The aromatic protons couple to the aromatic carbons (protonated) and C[1’-1]


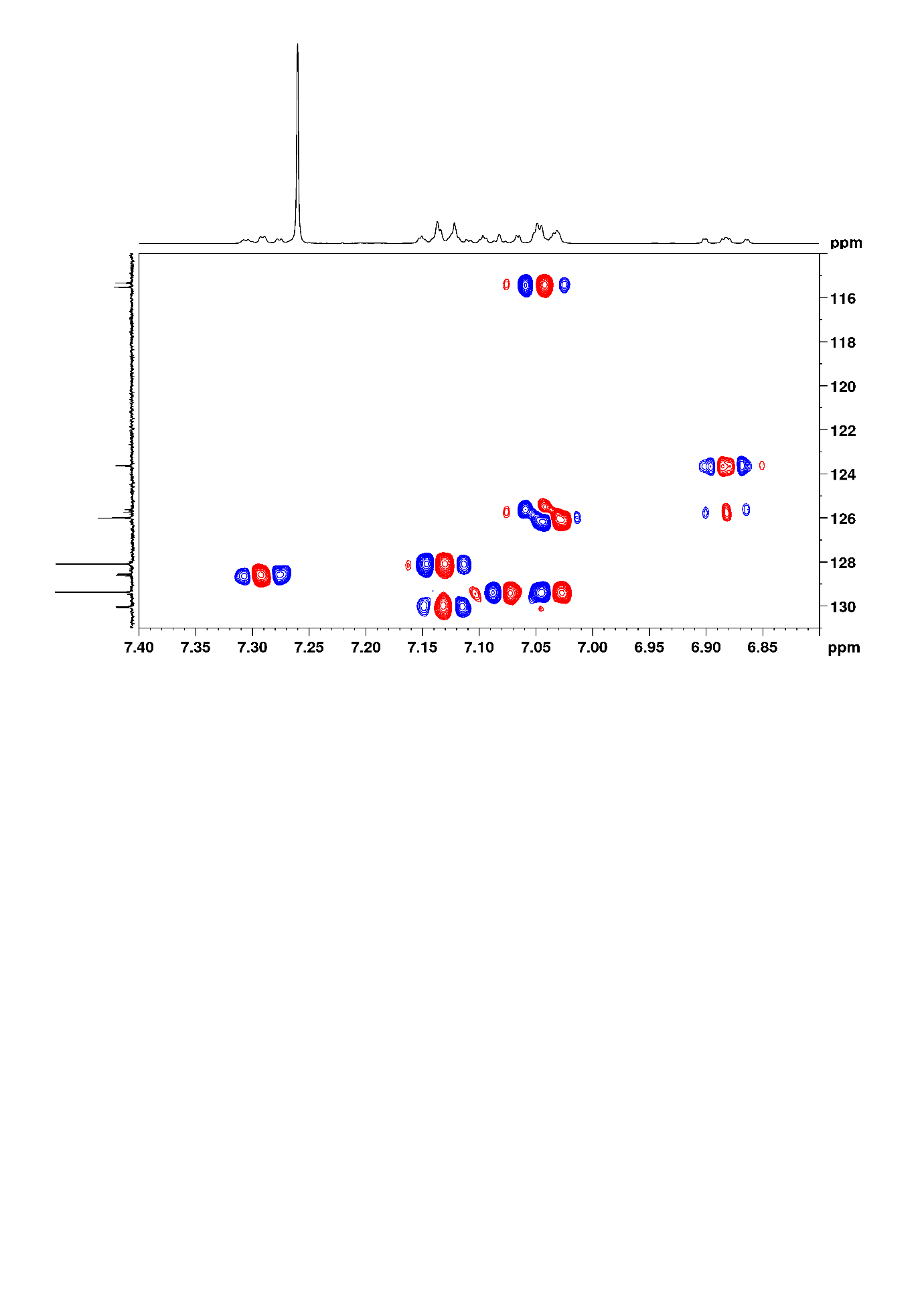


1’-6{1’-4}

1’-4{1’-6}

2’-3,5{2’-5,3}

1’-5{1’-1}

2’-2,6{2’-4}

1’-3{1’-1}

1’-3{1’-5}

1’-5{1’-3}

1. The aromatic protons couple to the remaining quaternary aromatic carbons


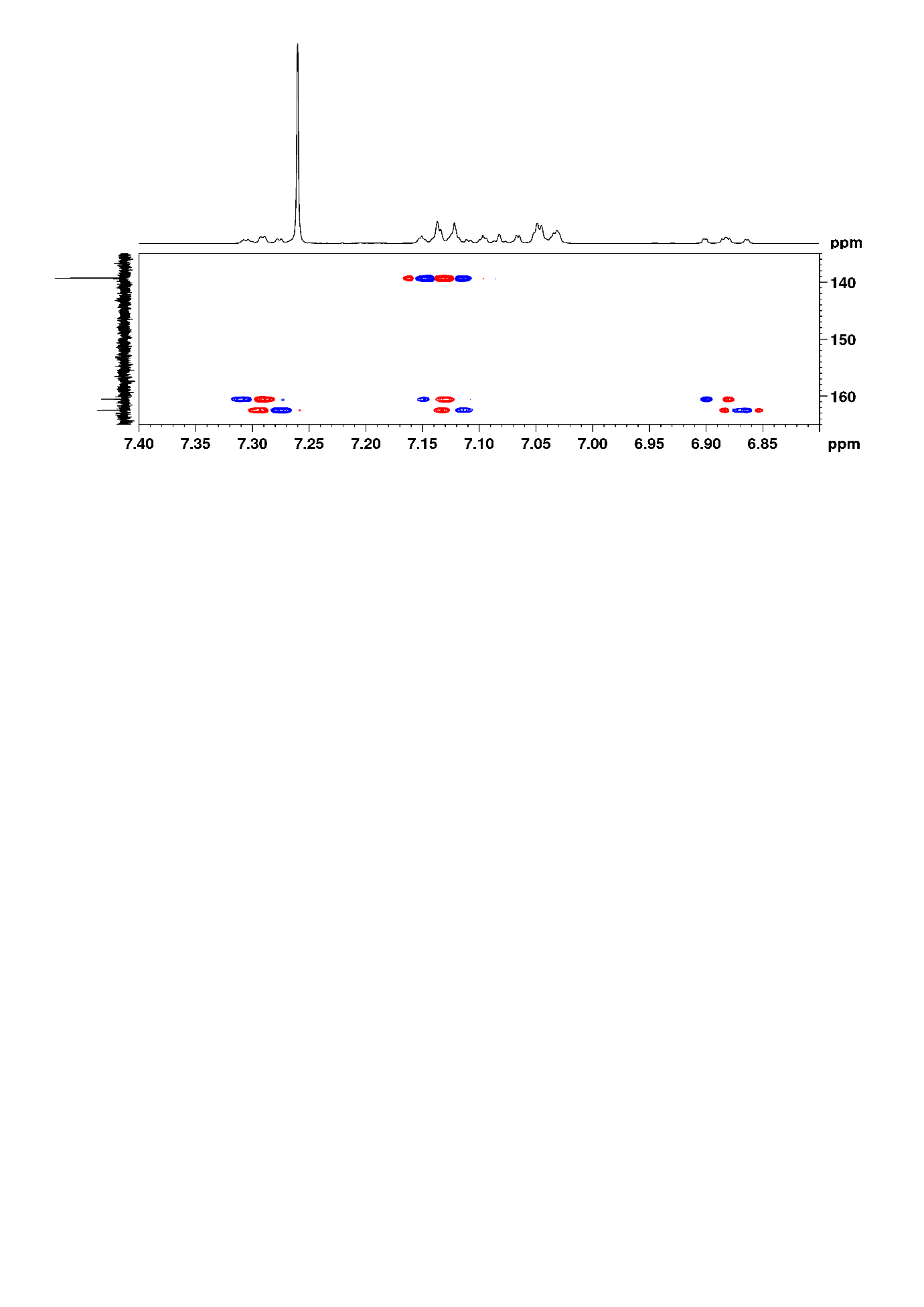


2’-3,5{2’-1}

1’-3{1’-2}

1’-4{1’-2}

1’-6{1’-2}

1. The aromatic protons couple to the aliphatic carbons


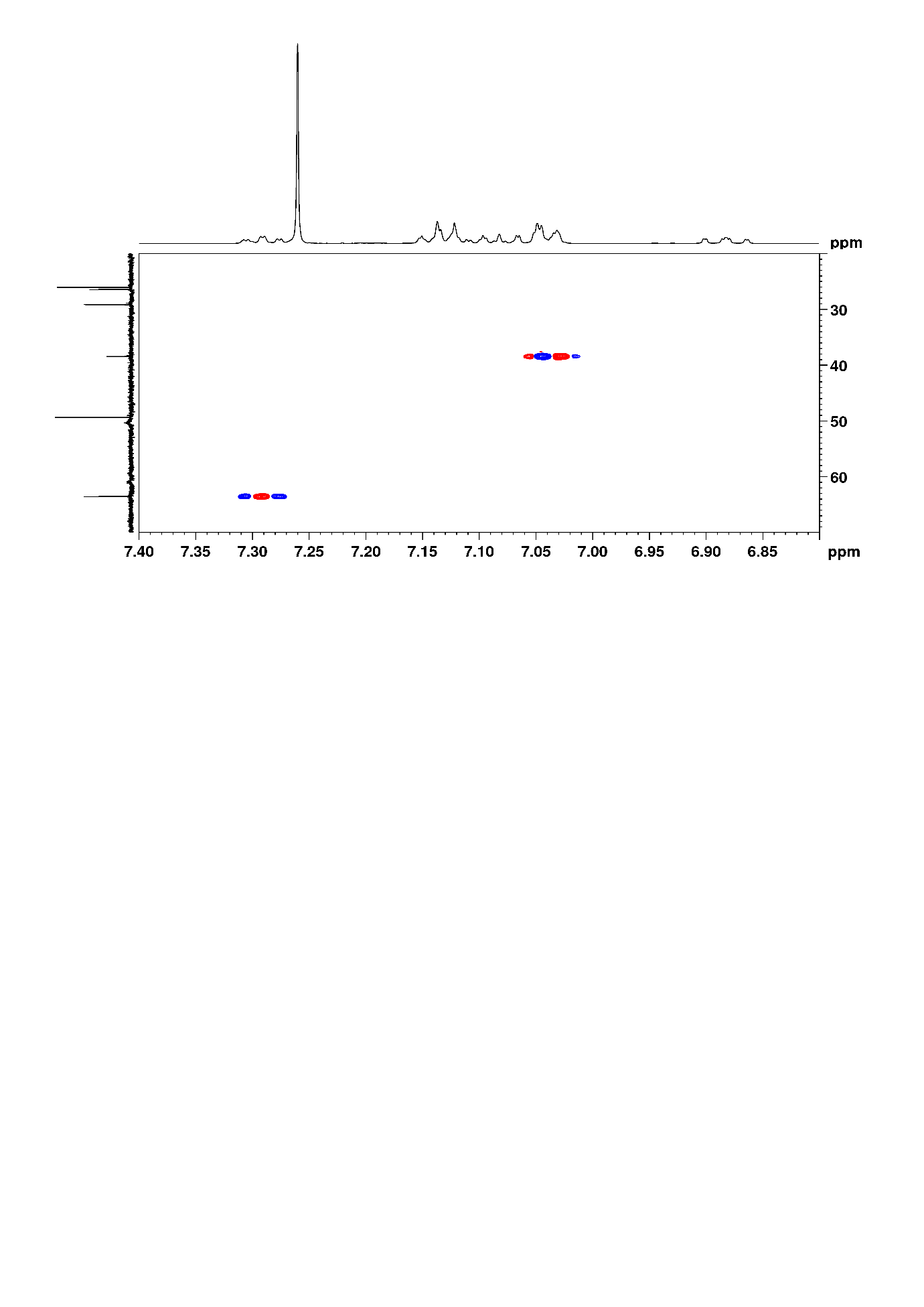


1’-6{1’}

2’-2,6{2’}

1. The aliphatic protons couple to the aliphatic carbons


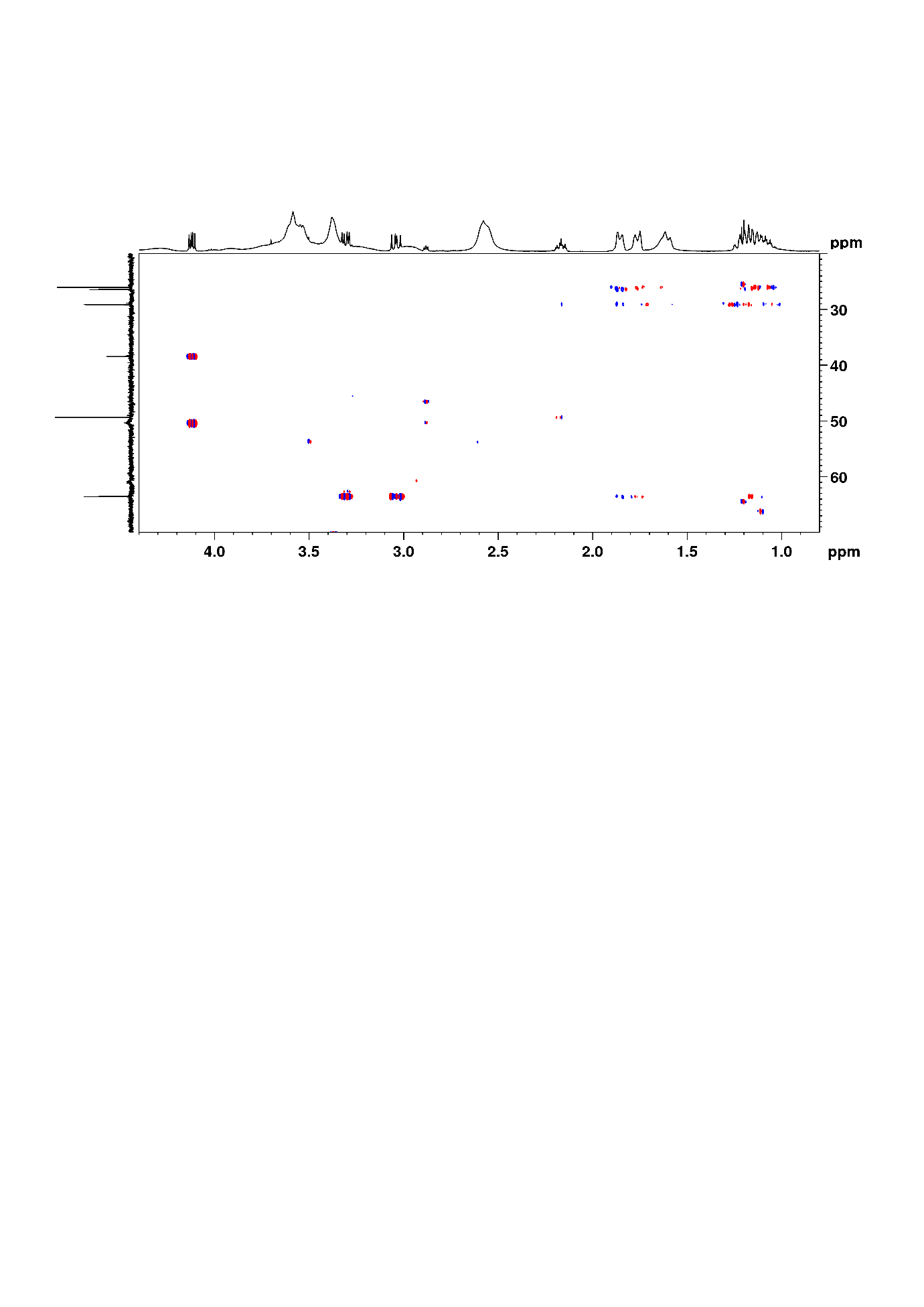


1’’{2,6}

1’{3,5}

2’{1’}

1’{2’}

1. The aliphatic protons couple to the aromatic carbons


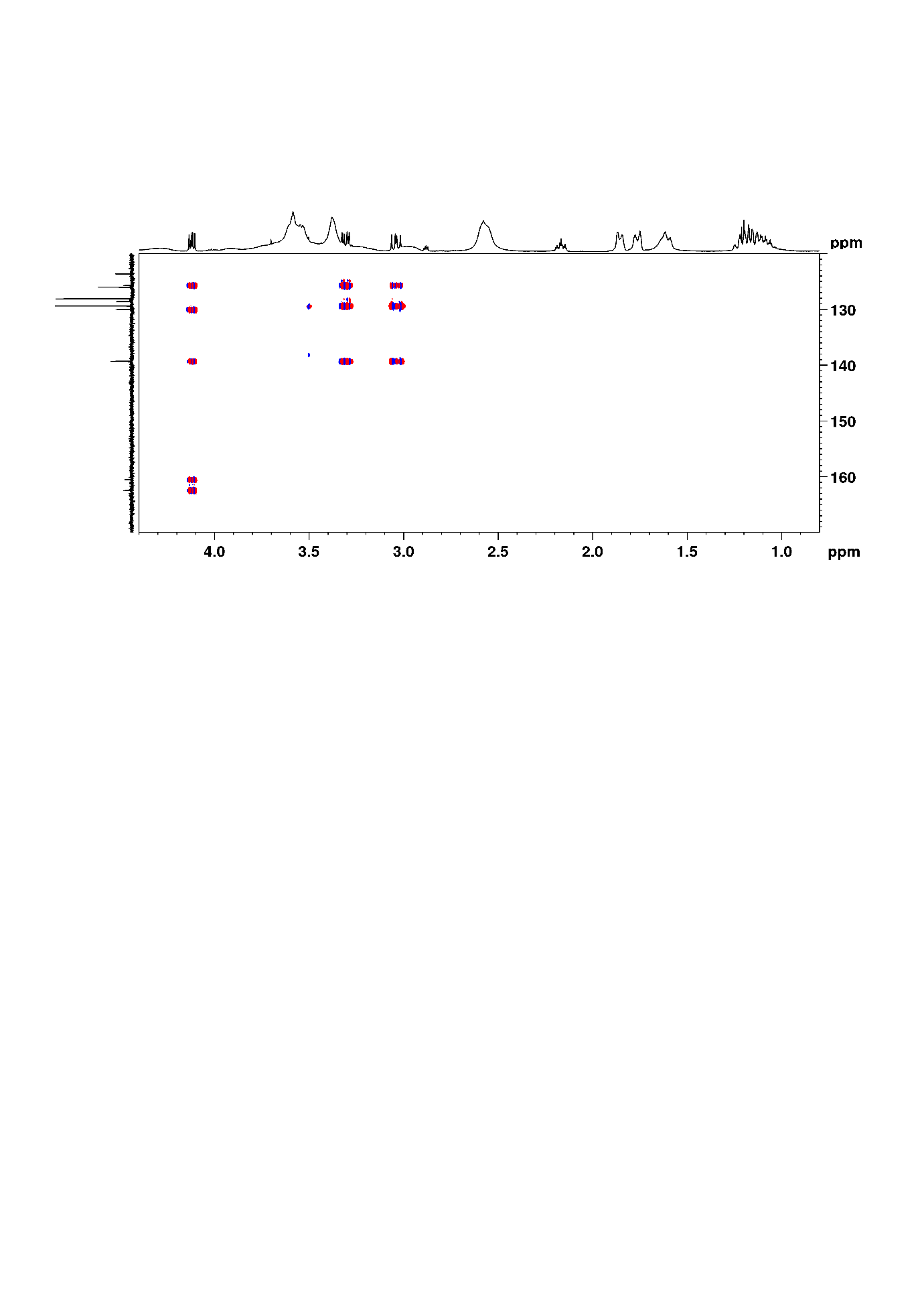


2’{2’-1}

2’{2’-1}

2’{2’-1}

1’{1’-2}

1’{1’-1}

1’{1’-6}

1’{2’-1}

1. Expansion of the ^1^H{^1^H} NOESY spectrum of the seized sample


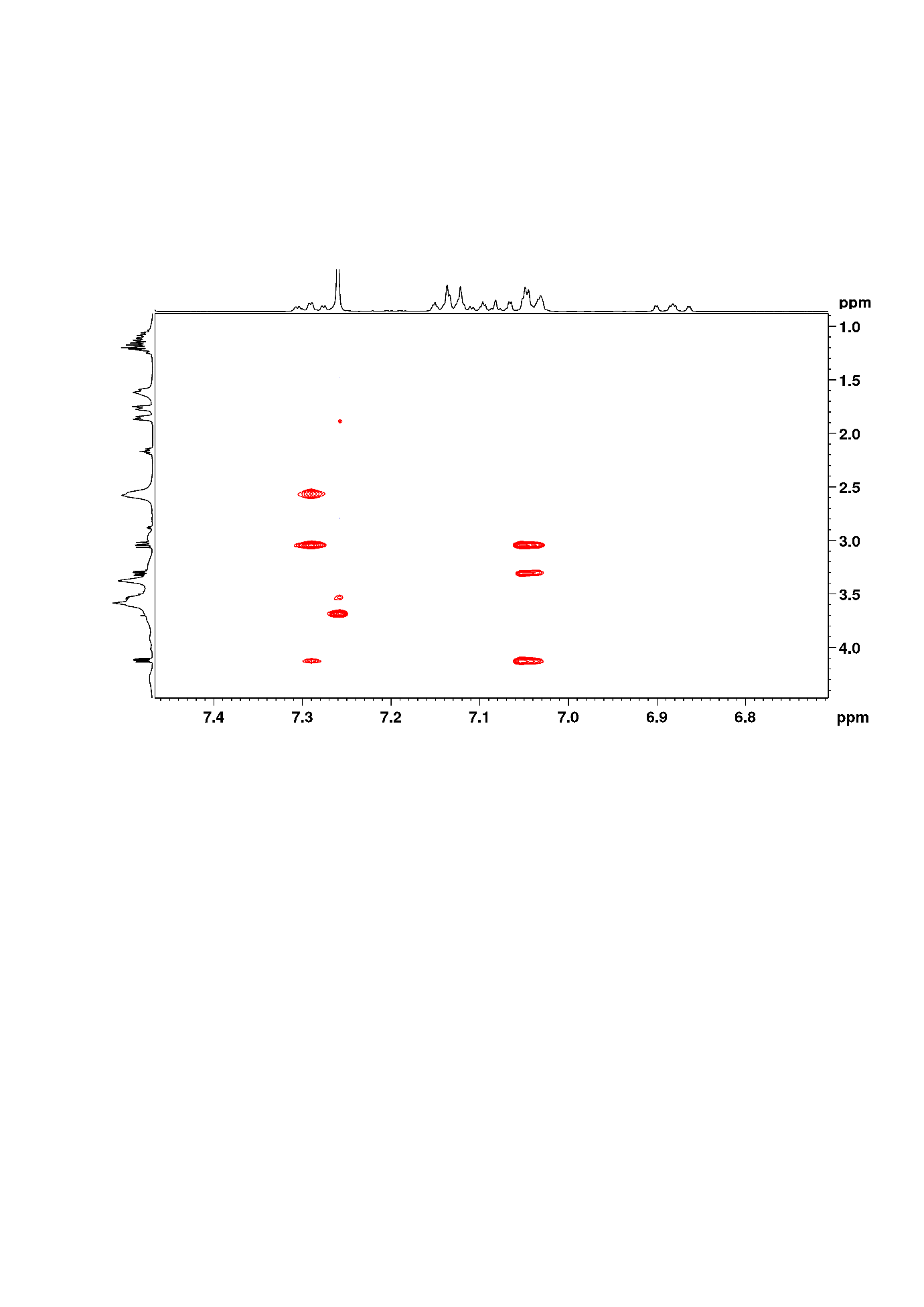


2’-2,6{1’}

2’-2,6{2’}

2’-2,6{2’}

1’-6{3,5}

1’-6{2’}

1’-6{1’}

1. ^1^H{^19^F} HOESY spectrum of the seized sample


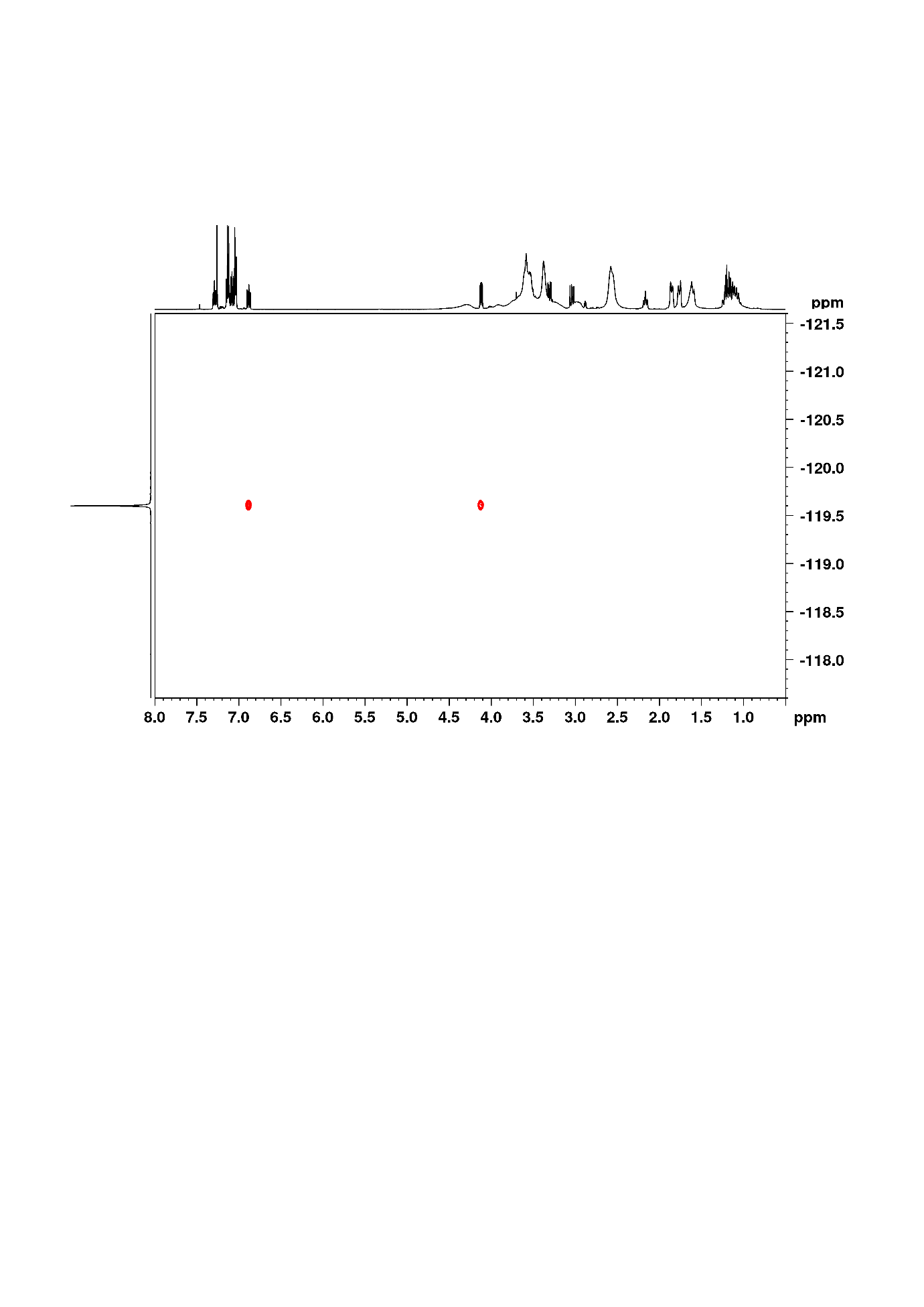


1’{1’-2}

1’-3{1’-2}

1. Raman spectra of the seized sample (red) and 2F-MT-45 reference standard (blue)

**
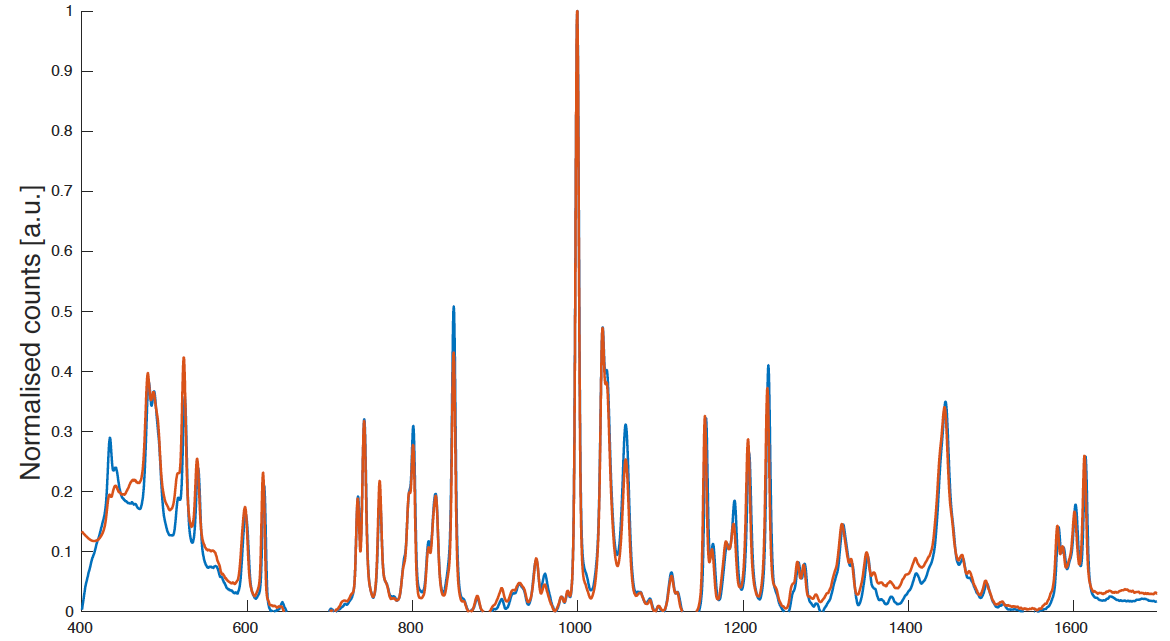
**

Raman Shift [1/cm]

**Section E MT-45 metabolism – mouse in vitro and iv vivo studies**

1. **Chromatographic profiles obtained following incubation of MT-45 with mouse hepatocytes after 60 min Metabolite labelling information can be found in Fig. 5 and Table 2.**

1. **Mouse urine collected 24 hours after MT-45 exposure. Metabolite labelling information can be found in Fig. 5 and Table 2.**

**Section F MT-45 Metabolite Structural Elucidation (UPLC-QToF MS/MS spectra)**


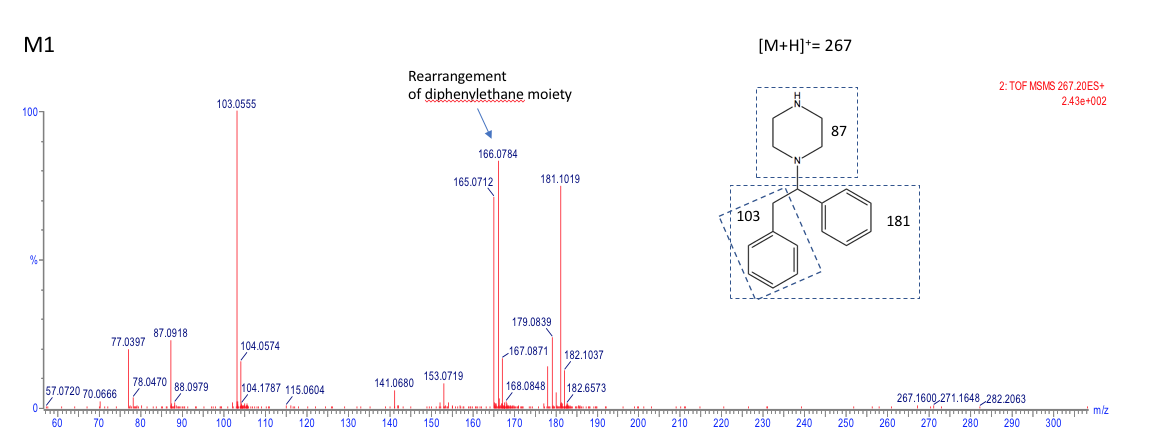


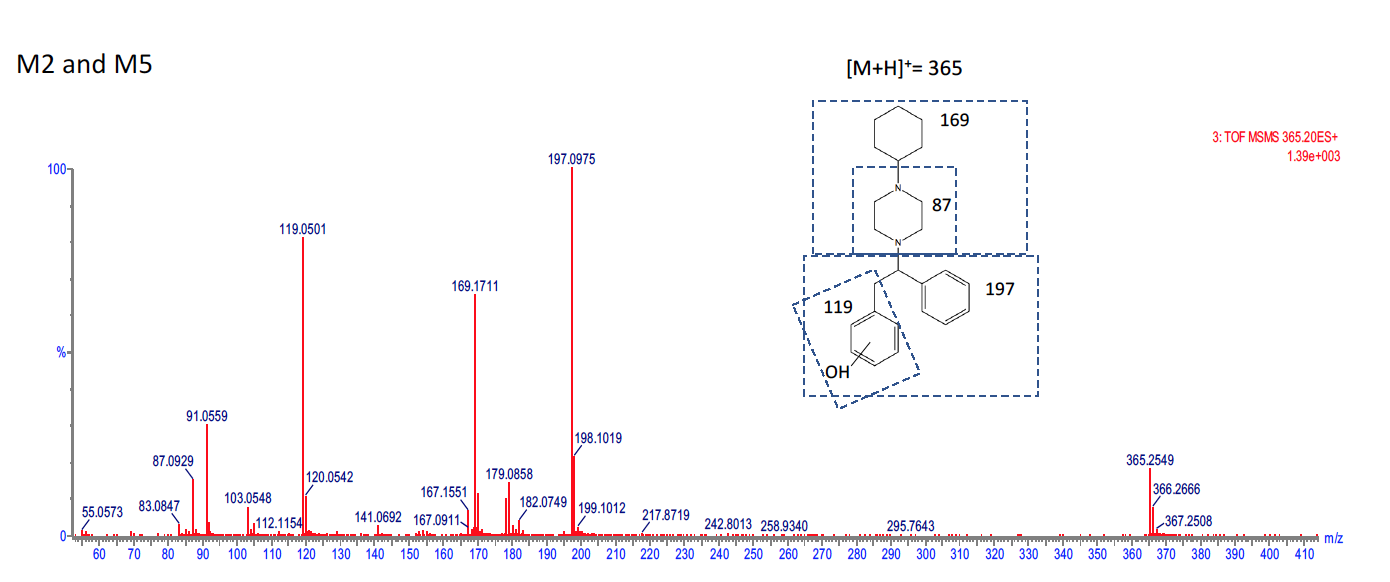


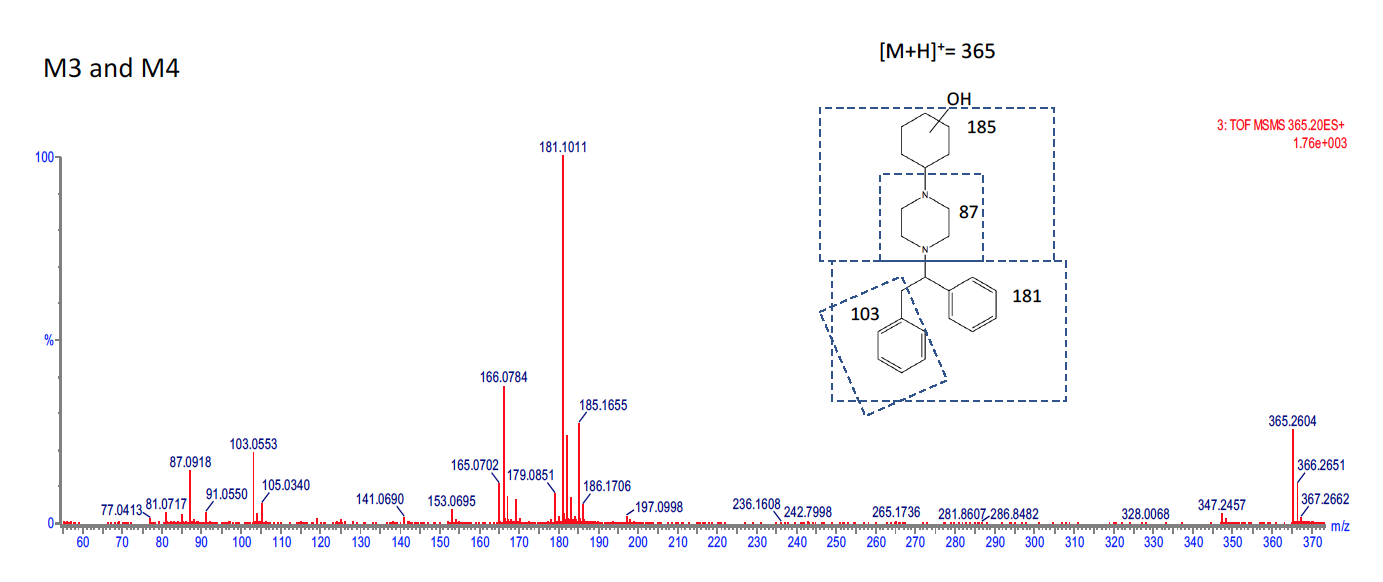


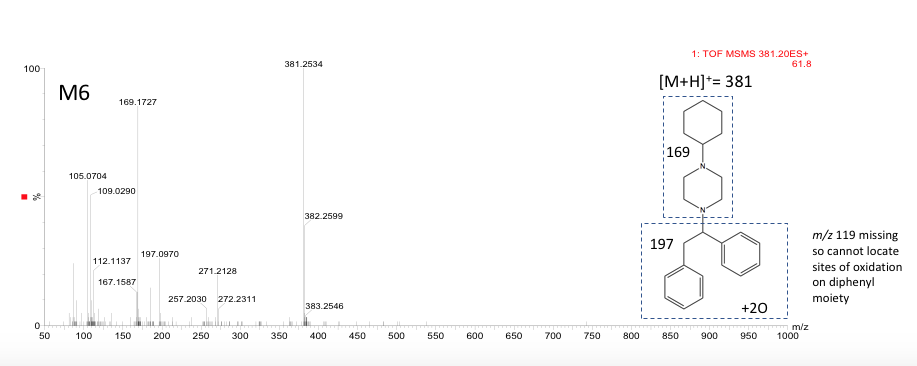


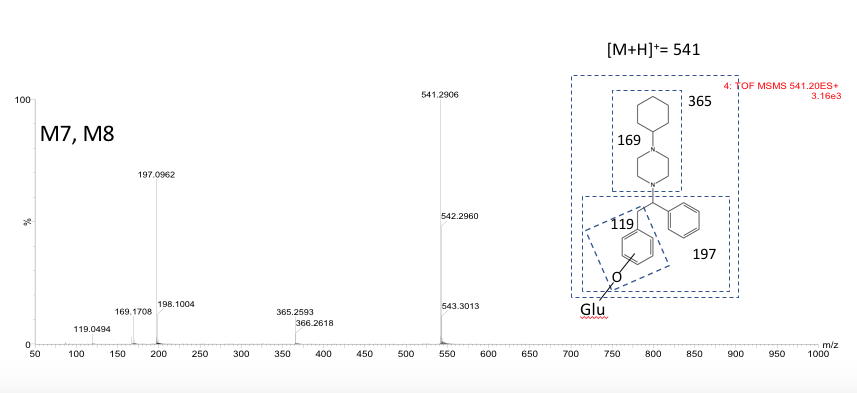


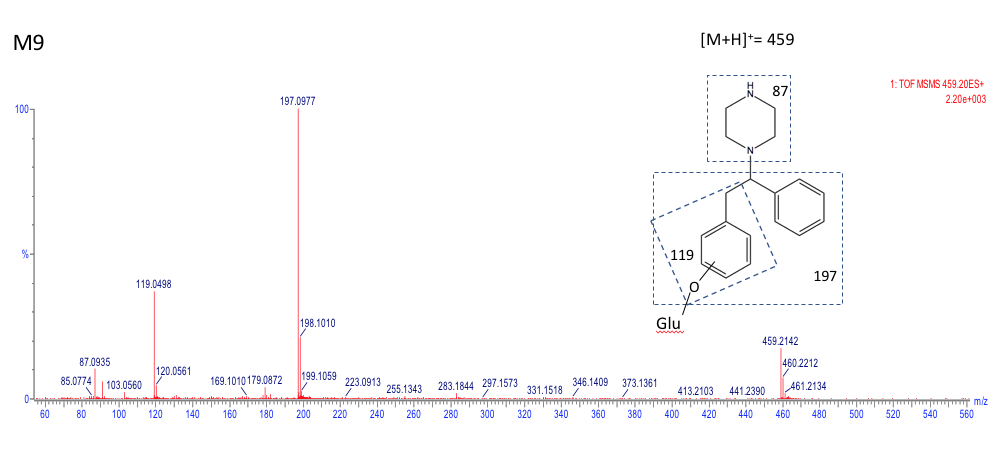


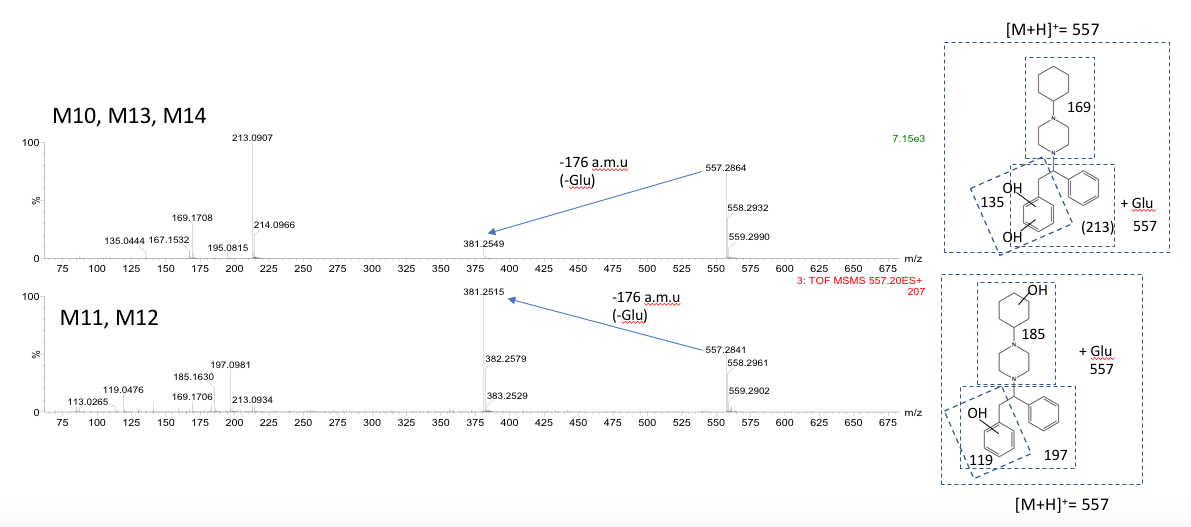


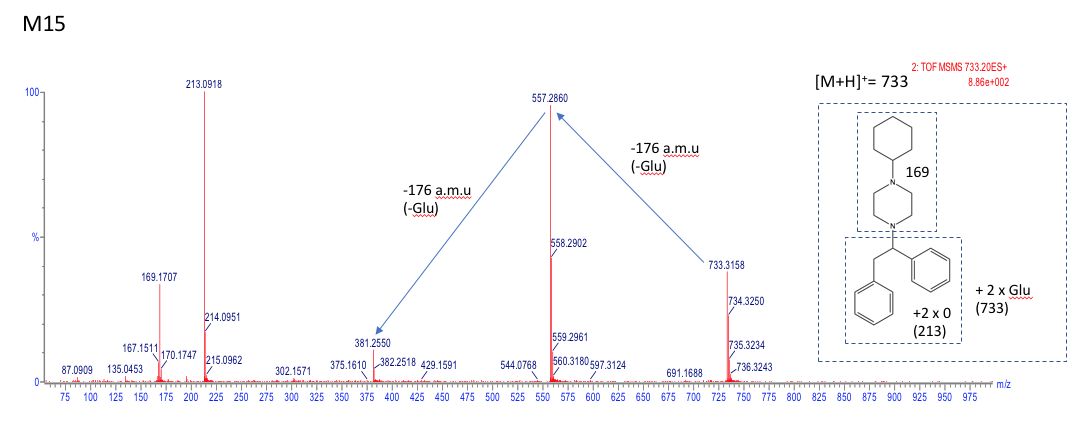


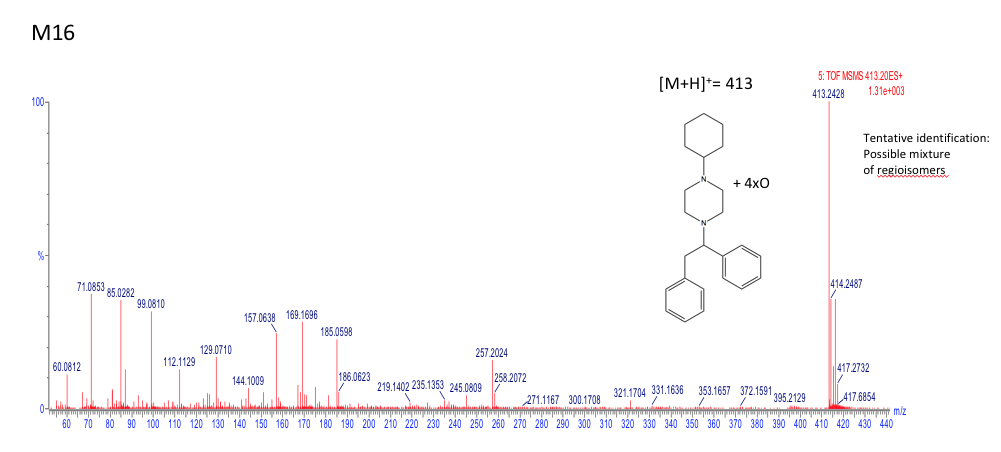


**PART G 2F- MT-45 Metabolite Structural Elucidation (UPLC-QToF MS/MS spectra)**

**
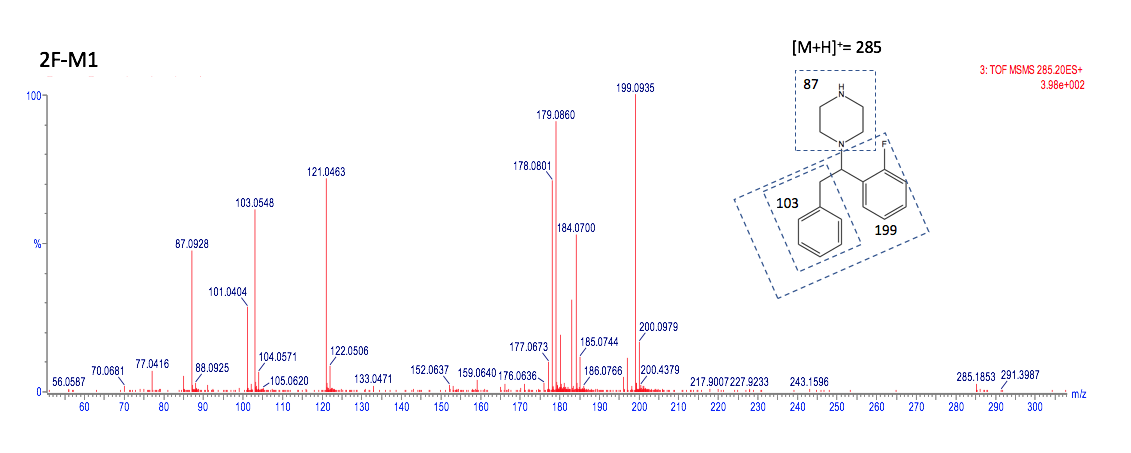
**

**
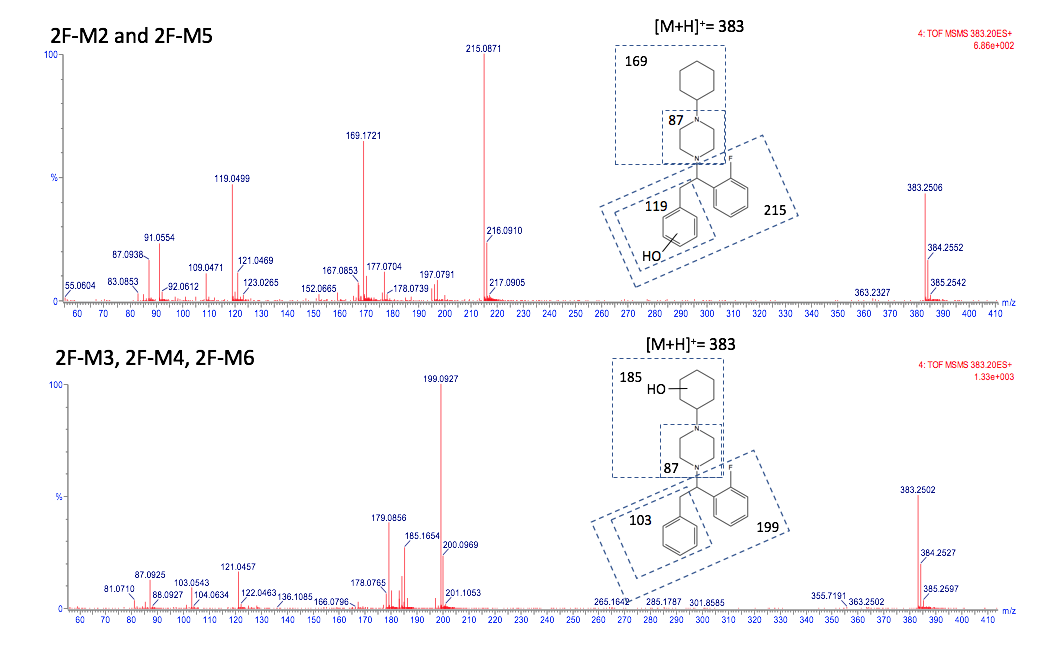
**

**
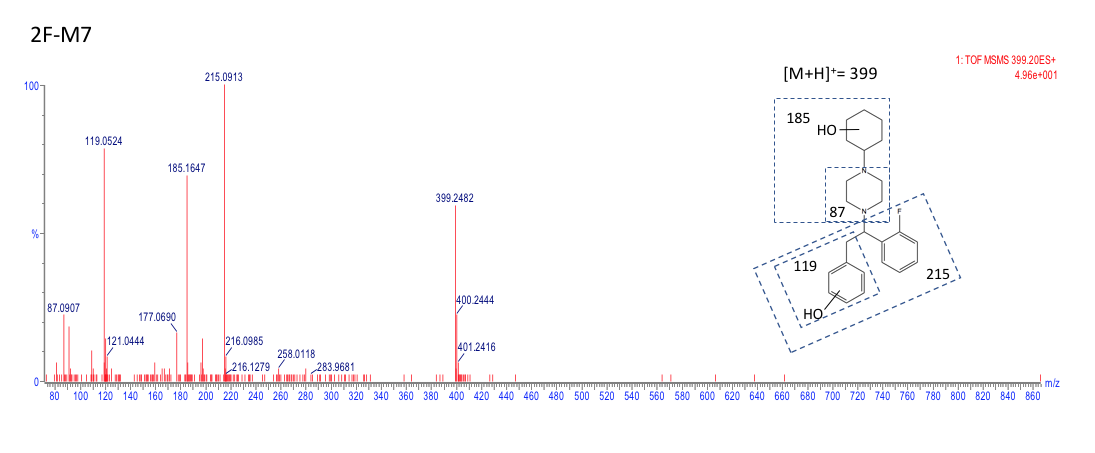
**

**
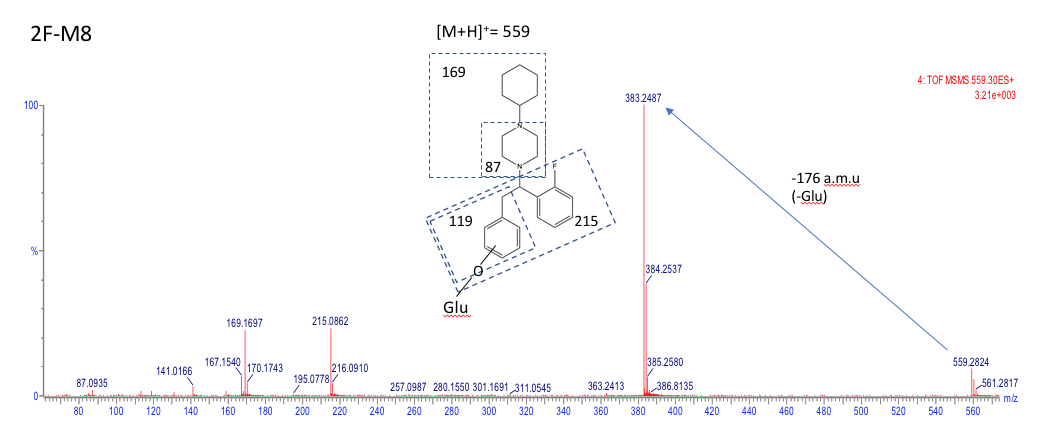
**

**
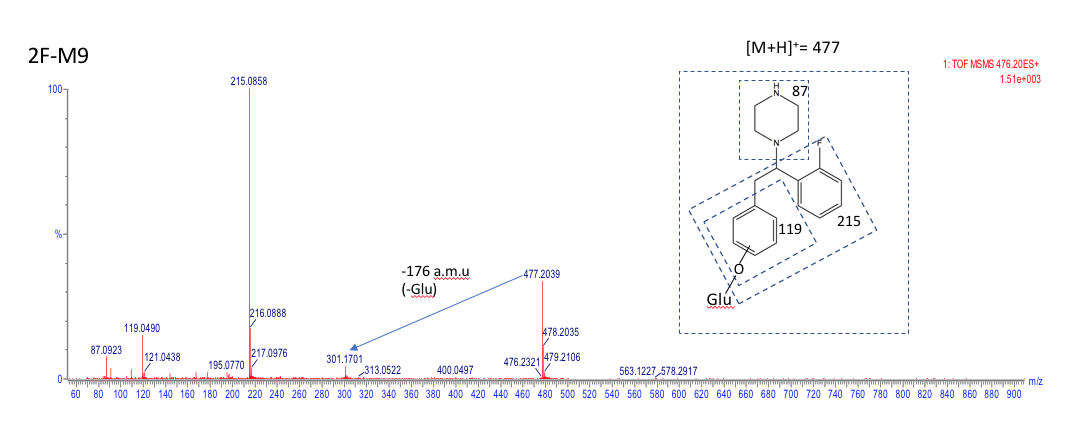
**

**
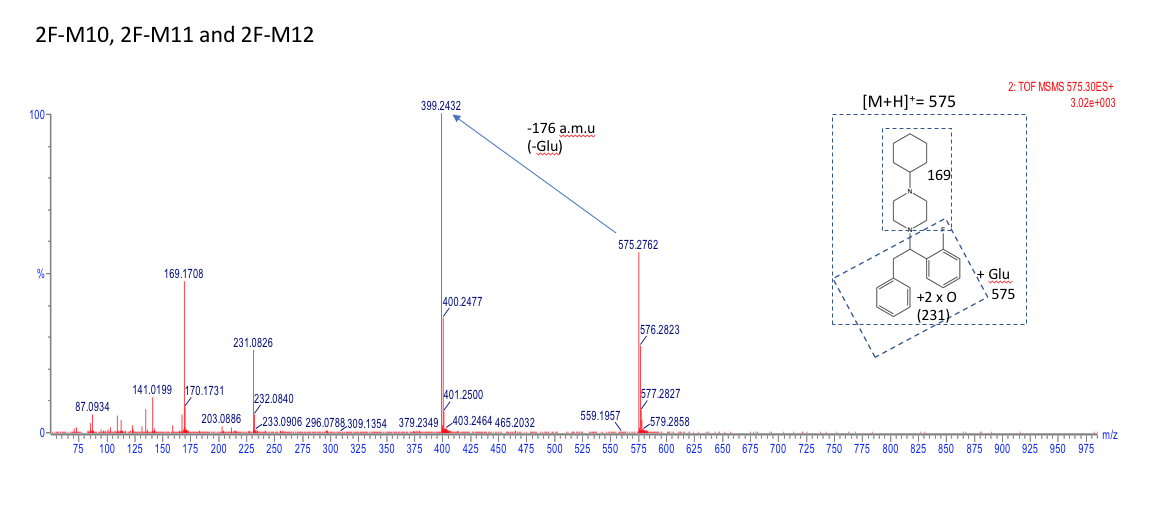
**
